# Fungicide use intensity influences the soil microbiome and links to fungal disease suppressiveness in amenity turfgrass

**DOI:** 10.1128/aem.01771-24

**Published:** 2025-02-21

**Authors:** Ming-Yi Chou, Apoorva Tarihalkar Patil, Daowen Huo, Qiwei Lei, Jenny Kao-Kniffin, Paul Koch

**Affiliations:** 1 Department of Plant Pathology, University of Wisconsin-Madison 312673 , Madison, Wisconsin, USA; 2 Department of Plant Biology, Rutgers University 124562 , New Brunswick, New Jersey, USA; 3 Horticulture Section, School of Integrative Plant Science, Cornell University 517685 , Ithaca, New York, USA; Royal Botanic Gardens, Surrey, United Kingdom

**Keywords:** turfgrass, suppressive soil, dollar spot, Clarireedia, microbiome

## Abstract

Disease-suppressive soils have been documented in many economically important crops, but not in turfgrass, one of the most intensively managed plant systems in the United States. Dollar spot, caused by the fungus *Clarireedia jacksonii*, is the most economically important disease of managed turfgrass and has historically been controlled through the intensive use of fungicides. However, previous anecdotal observations of lower dollar spot severity on golf courses with less intensive fungicide histories suggest that intensive fungicide usage may suppress microbial antagonism of pathogen activity. This study explored the suppressive activity of transplanted microbiomes against dollar spot from seven locations in the Midwestern U.S. and seven locations in the Northeastern U.S. with varying fungicide use histories. Creeping bentgrass was established in pots containing homogenized sterile potting mix and field soil and inoculated with *C. jacksonii* upon maturity. Bacterial and fungal communities of root-associated soil and phyllosphere were profiled with short-amplicon sequencing to investigate the microbial community associated with disease suppression. The results showed that plants grown in the transplanted soil microbiome collected from sites with lower fungicide intensities exhibited reduced disease severity. Plant growth-promoting and pathogen-antagonistic microbes may be responsible for disease suppression, but further validation is required. Additional least squares regression analysis of the fungicides used at each location suggested that contact fungicides such as chlorothalonil and fluazinam had a greater influence on the microbiome disease suppressiveness than penetrant fungicides. Potential organisms antagonistic to *Clarireedia* were identified in the subsequent amplicon sequencing analysis, but further characterization and validation are required.

**IMPORTANCE:**

Given the current reliance on fungicides for plant disease control, this research provides new insights into the potential non-target effects of repeated fungicide usage on disease-suppressive soils. It also indicates that intensive fungicide usage can decrease the activity of beneficial soil microbes and lead to a more disease conducive microbial environment in turfgrass. The results from this study can be used to identify more sustainable disease management strategies for a variety of economically important and intensively managed pathosystems. Understanding the factors that facilitate disease-suppressive soils will contribute to more sustainable plant protection practices.

## INTRODUCTION

Disease-suppressive soils have been of great interest for decades for their ability to suppress plant diseases without the intensive use of chemical inputs (1, 2). Suppressive soils have been identified for numerous economically important crops such as potatoes, wheat, strawberries, and bananas (3–6). According to the specificity and mechanism of the disease suppression, disease-suppressive soils are commonly classified as either general or specific (7). Specific suppression gains its suppressiveness from population-level antagonistic microbes against one or a small number of plant pathogens, whereas general suppression is derived from a complex interaction of diverse microbial taxa that is often suppressive to a broader range of plant pathogens (8). The formation of specific suppressive soils is commonly attributed to the coevolution of selective beneficial microbes, particularly those that inhibit pathogenic growth or development (7). For example, enrichment of fluorescent *Pseudomonas* spp. that produces the antifungal metabolite 2,4-diacetylphloroglucinol in the wheat rhizosphere after long-term monoculture led to the suppression of take-all of wheat (*Gaeumannomyces graminis* var. *tritici*) (9, 10).

Disease-suppressive soils can be induced and modulated through management practices, such as long-term monoculture, crop rotation, fertilization, inoculation of pathogen-antagonistic microbes, and soil amendment application (9, 11–13). The key to management impact on disease suppressiveness lies in the manipulation of the microbial association and dynamics surrounding the host plant(s) (8, 14). Fungicide applications are among the most common disease control methods but their impact on disease-suppressive soils has not been examined for the potential effects on disease-suppressive soil induction. Given the impact that fungicides have on the plant and soil microbiome, it is likely that fungicide usage plays a critical role in disease-suppressive soil formation (15–19). The turfgrass agroecological system is one of the most widespread and intensively managed plant systems in the U.S. and serves as an excellent model to study the roles of pesticide usage on microbe-plant-pathogen interactions and the resulting disease suppressiveness as they are perennial and constantly challenged by diseases.

Unlike many other economically important crops, disease-suppressive soils have not been documented in turfgrass, one of the most intensively managed plant systems in the U.S. Dollar spot is caused by the fungal pathogen *Clarireedia* spp. and is the most economically important disease of amenity turfgrass in temperate climates (20, 21). The fungus causes roughly circular patches of tan or brown turf 2–5 cm in diameter that results in a largely unplayable recreational surface (22). Host resistance to dollar spot among cultivars of creeping bentgrass (*Agrostis stolonifera*) exists but has not been widely implemented as a control strategy, and cultural practices do not typically provide commercially acceptable levels of dollar spot control (23, 24). This has resulted in more fungicides being used to suppress dollar spot than any other disease of golf course turfgrass (20). This heavy reliance on fungicides for acceptable control has resulted in the widespread development of fungicide resistance (25), economic hardship for many golf facilities (26), and concern over human and environmental contamination (27, 28). These concerns make the development of more sustainable dollar spot management strategies an important aspect of improving the overall sustainability of golf course management.

Decreased dollar spot severity on multiple golf courses following the conversion to reduced-fungicide disease management programs was observed. However, these observations remained anecdotal and the lack empirical investigations, indicating that more research is needed in this area. Furthermore, there has been strong interest in implementing biological control of *Clarireedia* using commercially available antagonistic microbes; however, success in the field has been lacking (29–33). Modulating the *in situ* microbiome to induce disease-suppressive soils requires comprehensive foundational knowledge of the amenity turfgrass microbiome and the impacts that various management practices can have. Several studies have examined the impact of management practices on the turfgrass microbiome. Amending turfgrass soil with different forms of carbon (C) and nitrogen (N) inputs stimulated short-term pulse of enzyme activities and microbial community spikes related to C and N cycling and respiration (34). However, Stacey et al. (35) found no shift in the turfgrass soil microbiome in a 2-year compost amendment experiment. Doherty and Roberts (36) reported that the fungicides propamocarb, fosetyl-Al, and cyazofamid did not significantly impact the rhizosphere bacterial diversity of creeping bentgrass in a 2-year field study. Rhizosphere bacterial communities associated with turf grown in higher soil iron content were found to significantly alter dollar spot development and severity (37). This last finding suggests that turfgrass microbial communities play an important role in the development of dollar spot, which may indicate that disease-suppressive soils are a plausible method for suppressing dollar spot. It is also important to note that these studies are relatively short term (< 3 years); hence, more investigations on the microbiome effect as a function of management practices are warranted.

This study sampled eight golf courses from the Midwestern and Northeastern U.S. with self-reported levels of high or low pesticide-use intensity. Soils from agricultural, prairie, and forest sites were also sampled in both geographic regions to add additional sites of varying management intensity for comparison to turfgrass. Next, the connection between dollar spot suppression, past fungicide use intensity, and the microbial factors associated with disease suppression were investigated. To accomplish this, turfgrass was established in a controlled environment in potting media with soil collected from each of the sites described above. The rhizosphere and phyllosphere microbiomes were profiled using high-throughput amplicon sequencing both prior to and after inoculation with the dollar spot fungus. We hypothesized that past fungicide use intensity would impact the natural disease suppressiveness of the soil, with important implications for the biological management of dollar spot and numerous other diseases in both turfgrass and other cropping systems.

## MATERIALS AND METHODS

### Sample collection, experimental design, and potting preparation

Field soil was collected between Aug 27 and Nov 1, 2019, from 14 sites in the Midwest (MW) and Northeast (NE) of the U.S. Soil was sampled from four golf courses, one agricultural field, one prairie, and one forest floor soil in each geographical region. Two golf courses in each region were identified as low fungicide intensity, and two were identified as high fungicide intensity based on prior knowledge of their disease management programs (Table 1). The high intensity programs were defined as additive programs built upon calendar-based fungicide applications, whereas low intensity programs were defined as reductive programs using decision support tools such as disease predictive models or simply had minimal or no fungicide options due to local regulations. Soils were sampled using a 2.75 cm diameter soil probe to a depth of 10 cm. Five soil cores were sampled from each site randomly within a 0.8 m^2^ area in the center of the fairway that represents the medium disease pressure per managers’ observation. For each golf course site, soil cores were sampled from creeping bentgrass (*Agrostis stolonifera*) fairways. The soil cores from MW were collected by the authors and stored at −80°C within 3 h of sampling, and the soil cores from the NE were sampled by the field managers on-site, individually wrapped in aluminum foil, and shipped overnight to Madison, WI and immediately stored at −80°C upon receipt.

**TABLE 1:** Sample description and the superintendent reported nitrogen and fungicide application rate in 2019

Name	Location	N quantity (kg/ha)	Fungicide application (a.i.-times/y)	Fungicide quantity (g a.i./m^2^)	Description
Control	–^*a*^	–	–	–	Sterile potting mix
MW-Ag	WI	0.00	0	0.00	Corn field
MW-Forest	WI	0.00	0	0.00	Forest floor
MW-High1	WI	35.03	14	3.13	Fairway of private golf course
MW-High2	WI	61.03	13	1.91	Fairway of private golf course
MW-Prairie	WI	0.00	0	0.00	35 plus year unmanaged prairie
MW-Low1	IL	48.82	5	0.66	Fairway of private golf course
MW-Low2	WI	24.41	9	1.75	Fairway of private golf course
NE-Ag	NY	NA^*b*^	NA	NA	Apple orchard
NE-Forest	NY	0.00	0	0.00	Forest floor
NE-High1	NJ	58.59	36	6.1	Fairway of private golf course
NE-High2	NJ	82.51	22	6.43	Fairway of private golf course
NE-Prairie	NY	0.00	0	0.00	15 plus year unmanaged ecotone
NE-Low1	MA	134.27	0	0.00	Fairway of private golf course
NE-Low2	NY	89.81	6	0.85	Fairway of public golf course

^*a*^ – indicates not applicable.

^*b*^ NA indicates data not available.

“Penncross” creeping bentgrass seeds were surface sterilized by treating the seeds with chloroform gas overnight and exposing them to UV light at 253.7 nm for 2 h in a biosafety cabinet (SterilGard Model SG503A-HE, The Baker Company, Sanford, ME, USA). Surface-sterilized creeping bentgrass seeds were sown and germinated on water agar before transferring to the pots to reduce the likelihood of damping off (*Pythium* spp) diseases injuring the seedlings. Calcined clay (Turface MVP, Oldcastle APG, Atlanta, GA, USA) and sand were homogenized (50/50 vol/vol) and placed in 500 mL plastic pots. The potted media was sterilized by autoclaving three times for 60 min following placement in the pots. Soils from the field were added into the pots (5 mL/pot) with five biological replications per soil source and carefully homogenized with a sterilized spoon and aggressive shaking in closed containers along with control without field soil inoculation. A 0.5 cm layer of sterilized sand was applied on top of the transferred seedlings to keep the roots covered and avoid drying out. Each pot was wetted with sterile distilled water with a vaporizer before and after seedlings’ transfer. All the tools and containers used in contact with seed and soil were sterilized. The pots were then placed in a UVC-sterilized growth chamber (Model 136LLVL, Percival Scientific, Perry, IA, USA) at 20/18 °C day/night temperature and 40% humidity with 16 h light period for 2 months before pathogen inoculation. The turf was trimmed to a height of 1 cm with sterilized scissors and irrigated with sterilized ddH2O using a vaporizer at a rate of approximately 20 mL/pot every other day throughout the incubation prior to pathogen inoculation.

### Pathogen inoculation and disease assessment

Inoculum was prepared by transferring 1-week-old potato dextrose broth (PDB)-cultured *Clarireedia jacksonii* (strain 2F92-1 collected in Madison, WI (38)) hyphae onto sterilized rye grains and incubated for 10 days at 20°C in the dark. Each pot was inoculated by placing five *Clarireedia*-infested rye grains in the middle of each pot at a depth of 0.5 cm below the turf canopy to ensure good contact between rye grains and the turf canopy-soil interface. The incubation condition was adjusted to 28/20 °C day/night temperature and 75% humidity with a 16-h light period to encourage dollar spot development.

The disease development monitoring followed a similar procedure to Chou et al. (37, 37). Briefly, digital pictures were taken 30 cm vertically above the turf, and the pictures were analyzed with Fiji (39) to calculate the percentage of green pixels within the measured turf surface area (i.e., greenness). The measurements were taken every 48 h starting from the day of pathogen inoculation and continued until 16 days after pathogen inoculation (DAI) and once again on 20 DAI. Percent greenness was defined as the greenness of each pot compared with the baseline greenness (100%) of that same pot measured on the day of pathogen inoculation (0 DAI).

### Soil and phyllosphere sampling and DNA sequencing

Soil and phyllosphere samples were collected from each pot immediately prior to pathogen inoculation and again 20 DAI. For the soil samples, the soil was sampled from each pot using an 8 mm metal cork borer to a depth of 2.5 cm with two random soil cores taken and pooled at each sampling point. For the phyllosphere samples, turfgrass leaves were collected from each pot by trimming the turfgrass plants at 1 cm using sterilized scissors.

Microbial DNA was extracted with Qiagen DNeasy PowerSoil Pro Kits (Qiagen, Hilden, Germany), and phyllosphere DNA was extracted using a Maxwell® RSC Plant DNA Kit (Promega, Madison, WI, USA). The amplicon-sequencing libraries were prepared following a modified method from M. S. Cox et al. (40). Briefly, the extracted DNA from each sample was diluted to 20 ng/μL followed by the amplification of 16S V4 and ITS2 regions using barcoded primers as described in (40). Each 25 μL PCR reaction contained 5 μL of template DNA and 12.5 µL of NEB master mix and underwent cycling conditions of an initial 95°C for 5 min followed by 25 cycles of 15 s at 95°C, 30 s at 60°C, 30 s at 72°C, and then a final extension at72°C for 8 min before storing the amplicons at −20°C. The PCR reactions for phyllosphere samples were the same as soil samples except the water was replaced with mitochondrial and chloroplast DNA clamps at 1 µM, and the cycling condition had an extra step of 68°C for 30 s (41). The amplicons were checked on 1.5% agarose gel, gel-extracted with Zymoclean Gel DNA Recovery Kit (Zymo Research, Irvine, CA, USA), normalized with Mag-Bind® EquiPure gDNA Normalization Kit (Omega Bio-Tek, Norcross, Georgia, USA), quantified with Qubit™ dsDNA HS assay (Thermo Fisher Scientific, Waltham, MA, USA), and equimolar pooled prior to sequencing on Illumina MiSeq (llumina, San Diego, CA, USA) system with 2 × 250 and 2 × 300 kits for 16S and ITS amplicons, respectively, at the University of Wisconsin, Madison Biotechnology Center.

### Bioinformatics and data analysis

Sequencing reads were demultiplexed using the default setting of bcl2fastq (llumina, San Diego, CA, USA), quality-filtered, and cleaned using the DADA2 (42) pipeline to generate amplicon sequence variants (ASVs) using R 4.0.2, and the taxonomic ranks were assigned with SILVA (v138) and UNITE (v8.2) reference databases (42–44). Only forward sequences were used for ITS sequences due to low quality reverse reads. Principal coordinates analysis (PCoA), ASV richness, Shannon diversity, and permutational multivariate analysis of variance (PERMANOVA) were performed with package “vegan” and “phyloseq” in R (45, 46). Evaluation of multivariate heteroscedasticity contribution to the community beta-diversity difference was conducted using “betadisper” in “vegan” to ensure the legitimacy of PERMANOVA results. Due to the large number of variables when using ASVs as predictors for dollar spot severity, a Random Forest machine learning algorithm was used to build the prediction model instead of conventional linear regression. The model optimization procedure followed the description outlined in Costa et al. (47). Briefly, Boruta feature selection was performed using the “Boruta” package with 100 iterations, 999 permutations, and 100 loops to identify the potential important variables, and then, the relative abundances of the consensus ASV were used in Random Forest (RF) modeling with the “randomForest” package in R (48, 49). Fungicide effect on the turfgrass greenness was then modeled using Spearman’s correlation and Partial Least Squares Regression with “pls” package in R (50) as the independent variables were all significantly correlated with each other and had a non-linear relationship with the greenness. The model p-value was derived by calculating the chance of the max r square values of the permutate models equal or greater than the minimum r-square value of the current model with 10,000 permutations. To strengthen the relationship between fungicide use intensity and microbial dollar spot suppression, counterfactual analysis was conducted according to Chernozhukov et al. (51) in R with package “Counterfactual” (52). This method makes inference on quantile effects derived from counterfactual distributions. Specifically, counterfactual analysis estimates conditional outcome redistribution and probabilities by modeling the relationship between the measured reference data and simulating the response variable shift using bootstrapping estimated variances when an artificially transformed variable redistribution is introduced. In our study, we artificially transformed the fungicide use quantity to 50% of the measured distribution mean as a counterfactual distribution and set the bootstrapping iterations to 100 times to conduct the analysis.

## RESULTS

### Disease development

**Fig 1:**
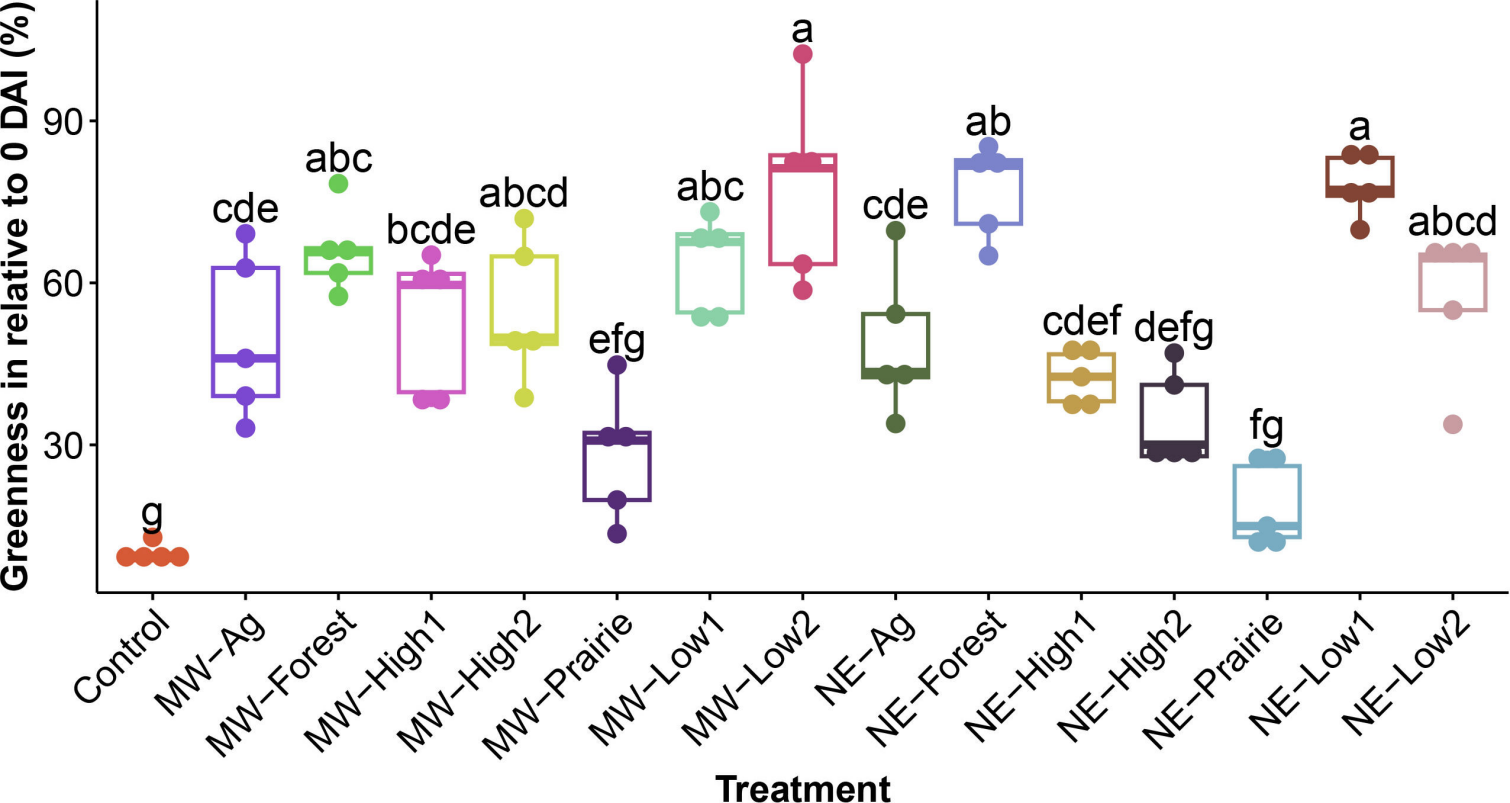
Boxplot showing turfgrass greenness as the indicator of dollar spot development on turfgrass established on varied field microbiome sources at 20 days post*-Clarireedia* inoculation and incubation under a conducive environment. The horizontal line in each box represents the median, and the upper and lower boundary of the box represents the first and third quartiles, respectively. Letters indicate the statistical difference yield from Tukey’s HSD for each day at α equals 0.05 where no sharing letters means significantly different.

Significant differences in dollar spot development, as measured by turf greenness, were observed (Fig. S1). Disease symptoms first appeared 6 DAI, and significant differences between treatments began to appear between 8 and 10 DAI. Although minor disease progression was observed post 16 DAI, the disease symptoms were most severe at the end of the experiment at 20 DAI (Fig. 1). By the end of the incubation, the non-treated control, which had no field soil added to the potting media, had the most severely diseased turf with less than 10% turf greenness. Other treatments exhibiting significant disease included NE-Prairie (18.8%), MW-Prairie (28.23%), and NE-High2 (34.64%). The treatments exhibiting the least amount of disease were NE-Low1 (78.14%), followed by MW-Low2 (77.87%), and NE-Forest (77.13%).

### Microbial richness and diversity

**Fig 2:**
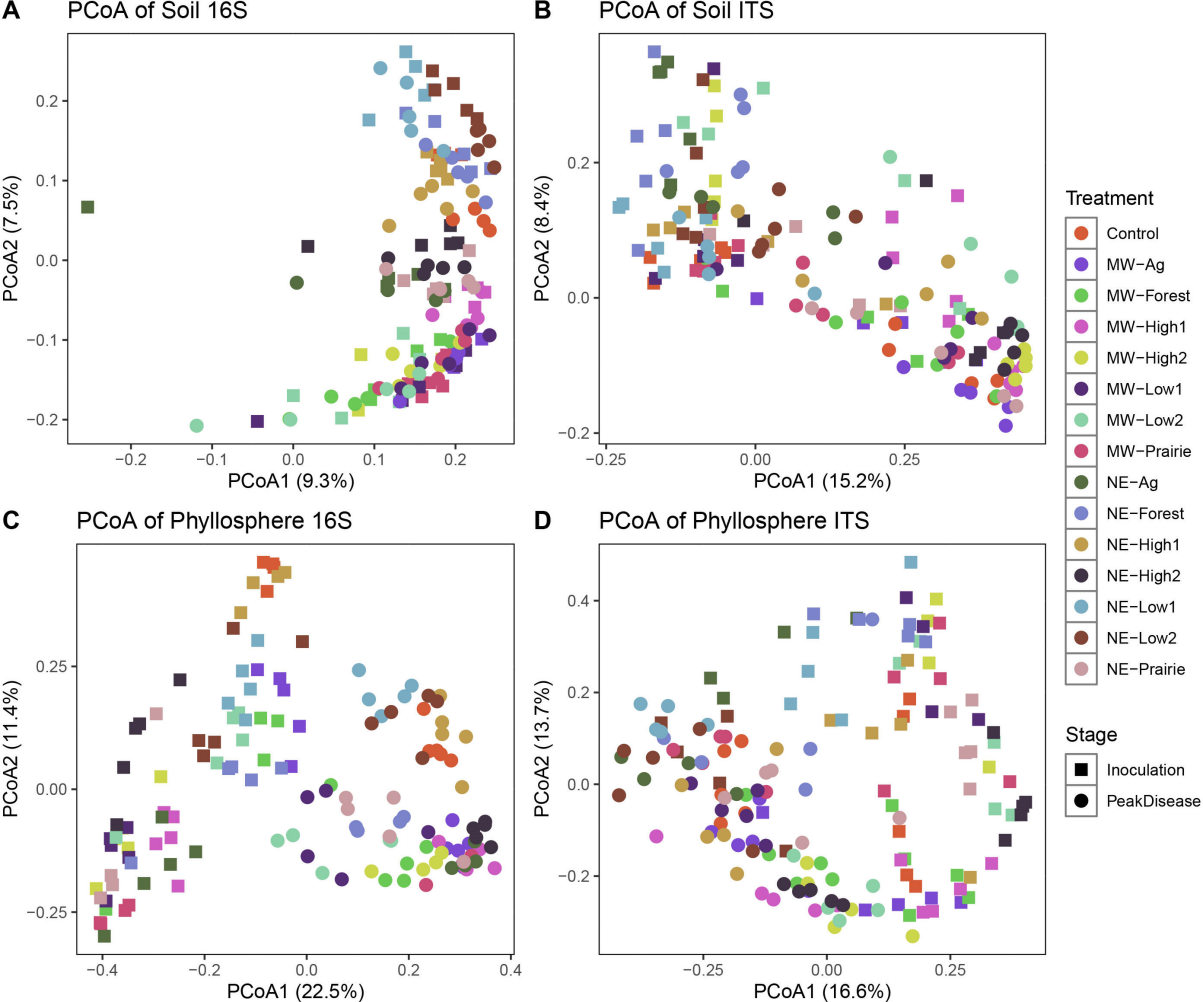
PCoA of microbiome associated with turfgrass grown with field microbiome transplantation presented with top two dimensions that explained most variances for each sample type including rhizosphere soil (**A and B**), and phyllosphere (**C and D**). The shapes indicate the sample stages, and the colors indicate the treatments (field microbiome sources).

A total of 6,344,855 and 13,340,254 reads were yielded with an average of approximately 16,000 and 33,000 reads after initial quality filtering for 16S and ITS samples, respectively. Bacterial and fungal community composition was distinct among sample types (soil and phyllosphere) and sampling stage (pretransplant, immediately before *Clarireedia* inoculation, and at peak of disease) as visualized in the two-dimension PCoA with Bray-Curtis distance (Fig. S2a, S2b). When analyzing the soil and phyllosphere samples separately, sample clustering by treatment was clearly observed for both bacterial and fungal communities regardless of sampling stage (Fig. 2A through D). Notably, shifts in soil bacterial and fungal communities occurred for all treatments after field soil microbiome transplantation. Also, there seemed to be a clear phyllosphere microbial community difference among sampling stages (Fig. 2C and D). The visual observation was statistically confirmed by permutational multivariate analysis of variance (PERMANOVA) (Table 2) and paired-PERMANOVA (Table S2). For both bacterial and fungal communities, the sample type and stage, treatments, and interactions all significantly explained the microbiome variances (Table 2). For the soil community, sampling type and stage explained the most variance for bacterial communities (R^2^ = 0.25, *P* < 0.0001) followed by treatment (R^2^ = 0.153, *P* < 0.0001), whereas treatment effect was more prevalent in fungal communities (R^2^ = 0.197, *P* < 0.0001) than that of sampling type and stage (R^2^ = 0.145, *P* < 0.0001). For the phyllosphere, treatment always explained the most variance for bacterial (R^2^ = 0.299, *P* < 0.0001) and fungal (R^2^ = 0.318, *P* < 0.0001) communities. Although the read number variation across the samples was also a significant factor, the variance explained ranging from 2.1% to 11% was only a fraction of the treatment effects (15%–32%). The treatment effect was further validated with paired-PERMANOVA where almost all pairs across sample types and sampling time were significantly different in both bacterial and fungal communities with rare exceptions (Table S1). ASVs homogeneity test was conducted with beta-dispersion, and only significant differences were observed in fungal communities among different field soils, whereas other sample types and sampling stages were not significant across treatments (Table S2).

**TABLE 2:** PERMANOVA analyses for turf phyllosphere and rhizosphere soil bacterial and fungal communities^*a*^

	R^2^	Pr(>F)	*P* value
Overall 16S			
Reads	0.04838	1.00E-04	***^b^
TypeStage	0.24995	1.00E-04	***
Treatment	0.15293	1.00E-04	***
Reads:TypeStage	0.03253	1.00E-04	***
Reads:Treatment	0.04885	1.00E-04	***
TypeStage:Treatment	0.22674	1.00E-04	***
Reads:TypeStage:Treatment	0.06748	1.00E-04	***
Residuals	0.17314		
Overall ITS			
Reads	0.03713	1.00E-04	***
TypeStage	0.14481	1.00E-04	***
Treatment	0.1968	1.00E-04	***
Reads:TypeStage	0.03215	1.00E-04	***
Reads:Treatment	0.05584	1.00E-04	***
TypeStage:Treatment	0.21946	1.00E-04	***
Reads:TypeStage:Treatment	0.07556	1.00E-04	***
Residuals	0.23824		
Soil 16S			
Reads	0.04743	1.00E-04	***
Stage	0.14668	1.00E-04	***
Treatment	0.27599	1.00E-04	***
Reads:Stage	0.01523	1.00E-04	***
Reads:Treatment	0.06956	1.00E-04	***
Stage:Treatment	0.17918	1.00E-04	***
Reads:Stage:Treatment	0.05529	1.00E-04	***
Residuals	0.21064		
Soil ITS			
Reads	0.02177	0.0001	***
Stage	0.11756	0.0001	***
Treatment	0.23354	0.0001	***
Reads:Stage	0.01483	0.0001	***
Reads:Treatment	0.07179	0.0001	***
Stage:Treatment	0.20455	0.0001	***
Reads:Stage:Treatment	0.06396	0.0049	**
Residuals	0.272		
Phyllosphere 16S	R^2^	Pr(>F)	
Reads	0.08154	1.00E-04	***
Stage	0.15405	1.00E-04	***
Treatment	0.29923	1.00E-04	***
Reads:Stage	0.02079	1.00E-04	***
Reads:Treatment	0.09847	1.00E-04	***
Stage:Treatment	0.11546	1.00E-04	***
Reads:Stage:Treatment	0.0543	1.00E-04	***
Residuals	0.17616		
Phyllosphere ITS			
Reads	0.11228	1.00E-04	***
Stage	0.08958	1.00E-04	***
Treatment	0.31829	1.00E-04	***
Reads:Stage	0.0139	1.00E-04	***
Reads:Treatment	0.12777	1.00E-04	***
Stage:Treatment	0.07731	1.00E-04	***
Reads:Stage:Treatment	0.05182	1.00E-04	***
Residuals	0.20905		

^*a*^ Reads refer to number of reads after quality filtering for each sample, treatments represent different sources of field soil inocula, and TypeStage indicates the sample types including phyllosphere and rhizosphere soil as well as sampling stages including field inocula, pre-inoculation of pathogen, and peak of disease.

^*b*^ ***, statistical significance level with *P* value < 0.001.

**Fig 3:**
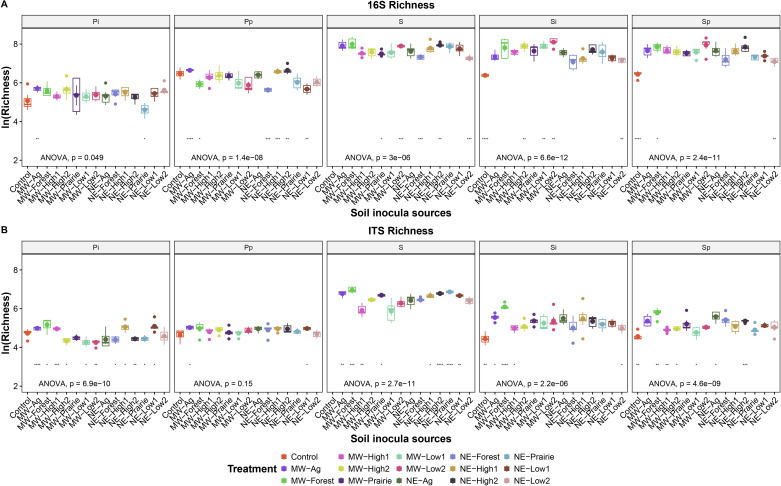
ASV richness for (**A**) bacterial and (**B**) fungal communities in turfgrass grown with different sources of transplanted field microbiomes. The asterisks indicate significant mean separation derived from *t*-test: *, *P*  <  0.05; **, *P*  <  0.01; ***, *P*  <  0.001; ****, *P*  <  0.0001. The panel headers indicate the sample types including phyllosphere (capital P) and root-associated soil (capital S) as well as sampling stages including field inocula (no lowercase designation), pre-inoculation of pathogen (lowercase i), and peak of disease (lowercase p).

Differences in microbiome α-diversity, measured as natural log richness and Shannon diversity index, between field soil inoculum were observed (Fig. 3). For bacterial richness, NE-Low2, NE-Forest, and MW-Prairie were among the lowest, and NE-High2 and MW-Low2 were among the highest (Fig. 3A). This trend generally held for bacteria in the soil at pathogen inoculation and became more even at peak disease. In contrast, the phyllosphere bacterial richness was even across the treatments for except NE-Prairie and MW-Ag but became more divergent at the peak of the disease. For fungal richness in the field soil inoculum, non-golf course soil from Midwest, NE-High1, NE-High2, NE-Low1, and NE Prairie had the highest richness, and the MW-High1 had the lowest (Fig. 3B). The soil fungal richness decreased after soil inoculation and turf establishment, but MW-Ag and MW-Forest were among the highest in richness and MW-High1 was among the lowest, which was similar to the bacterial samples. The soil fungal richness became slightly more divided among the treatments at the peak of disease compared with pathogen inoculation sampling, whereas the phyllosphere fungal richness spanned a narrower range across the treatments at the end of the experiment when the disease peaked. The potting media without soil had the lowest bacterial and fungal richness in the soil regardless of the sampling stage. A trend of dropping in soil microbial richness was observed after microbiome transplant, and diversity recovery was only observed in the soil bacterial community at peak disease but not in the fungal community (Fig. S3). Phyllosphere bacterial and fungal richness increased from pathogen inoculation to peak disease but remained lower than that of soil samples (Fig. S3). Shannon diversity generally showed a similar trend as the richness (Fig. S4).

### Identifying potential disease-suppressive predictor microbes

**Fig 4:**
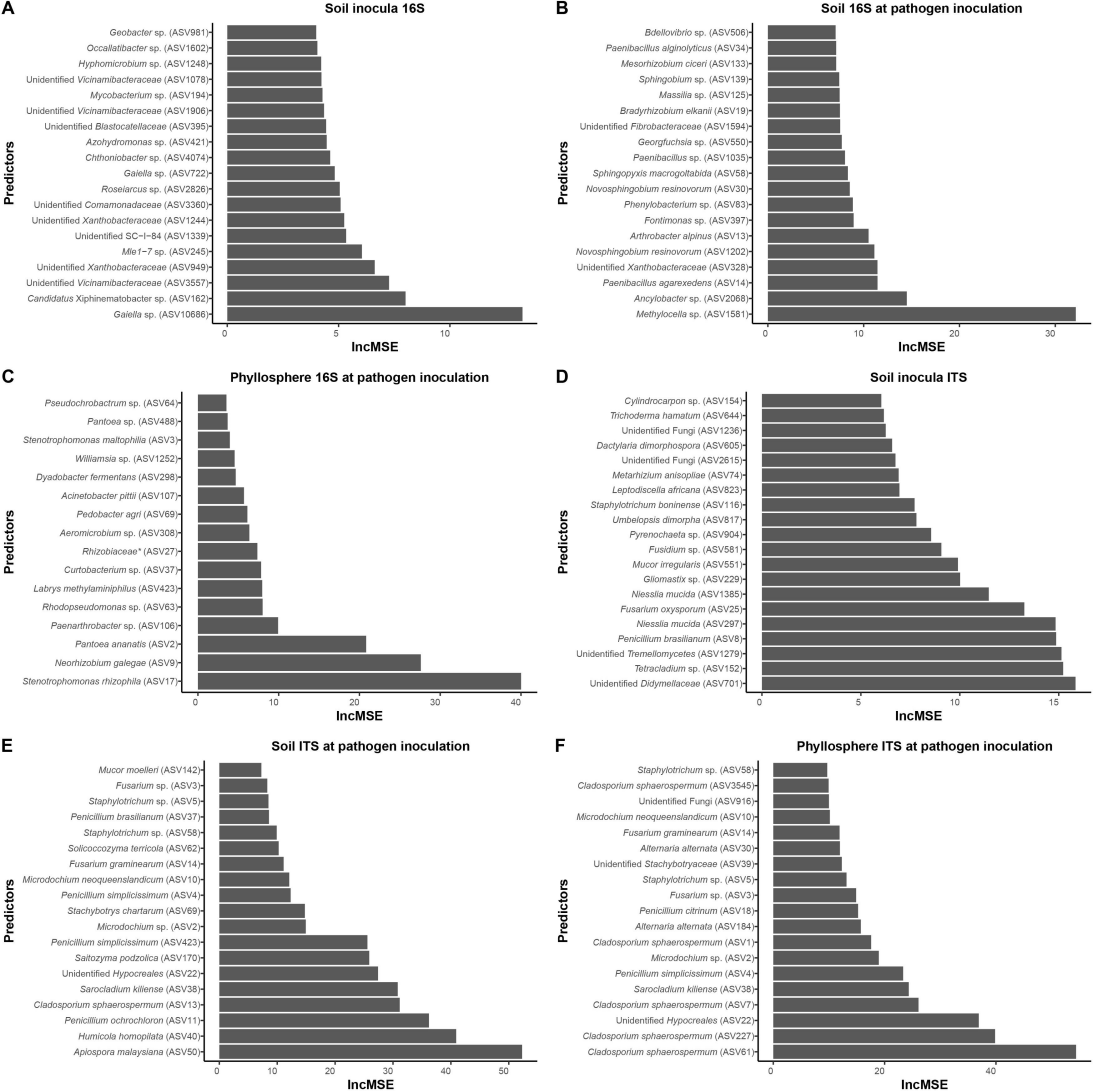
Important microbial predictors for the dollar spot suppressiveness selected by Random Forest models for bacteria (**A, B, and C**) and fungi (**D, E, and F**) from the transplanted microbiome inocula, rhizosphere soil, and phyllosphere at pathogen inoculation. IncMSE indicates an increase in mean square error, and the importance of the indicators increases following the increase in IncMSE.

Microbial taxa in the turf-associated microbiome showed different relative abundances across the treatments (Fig. S5 and S6). However, due to the complex microbial composition and large number of variables to model the microbial disease-suppressive relationship, a machine learning algorithm was used to decipher the association. In this model, ASVs were used as features to predict the turf greenness as an indicator of disease suppressiveness. Boruta was applied to select the relevant bacterial and fungal ASVs, and Random Forest was used to build a predictive model. Significant models were built with more than 60% variance explained by using either soil bacterial or fungal ASVs to predict disease suppressiveness, whereas models built with phyllosphere bacterial and fungal ASVs resulted in 25.15% and 53.82% variance explained, respectively (Fig. S7). All models were significant (model *P*-value < 0.05), suggesting non-random microbial assembly that was influenced by treatment. Top disease-suppressive bacterial and fungal predictors were selected and ranked by their increase in mean square error for each sample type and sampling time (Fig. 4). Distinctively effective predictor ASVs, as evaluated by increase of mean square error, were observed for each sample type and stage including the bacteria *Gaiella* sp., *Methylocella* sp., *Stenotrophomonas rhizophila*, *Neorhizobium galegae*, and *Pantoea ananatis* and the fungi *Apiospora malaysiana* and *Cladosporium sphaerospermum*.

For the RF model-selected important microbial predictors, correlation analyses were performed to associate their relative abundance and the dollar spot disease suppressiveness (Table 3). The RF model selected ASVs from samples at the time of pathogen inoculation and the results showed many significantly and positively correlated taxa, including the fungi *Microdochium neoqueenslandicum*, *Mucor moelleri, Saitozyma podzolica*, *Microdochium* sp., *Penicillium simplicissimum*, *Humicola homopilata*, and *Solicoccozyma terricola* (Table 3) and the bacteria *Mesorhizobium ciceri*, *Bradyrhizobium elkanii*, unidentified *Xanthobacteraceae*, and *Phenylobacterium* sp. (Table 4).

**TABLE 3:** Correlation test of relative abundances of random forest selected important fungal taxa from the turf rhizosphere soil prior to *Clarireedia* inoculation with turfgrass greenness after disease development, and field nitrogen and fungicide applications

	Greenness (20 DAI)		N quantity		Fungicide quantity		Fungicide frequency	
Fungal taxa	R	*P*-value	R	*P*-value	R	*P*-value	R	*P*-value
*Microdochium neoqueenslandicum*	0.18	0.01	0.23	0.00	−0.14	0.05	−0.15	0.05
*Penicillium ochrochloron*	−0.11	0.13	−0.15	0.05	−0.12	0.09	−0.13	0.06
*Cladosporium sphaerospermum*	−0.15	0.05	−0.10	0.23	−0.12	0.08	−0.11	0.10
*Fusarium graminearum*	−0.35	0.00	−0.42	0.00	−0.31	0.00	−0.30	0.00
*Mucor moelleri*	0.24	0.00	0.08	0.31	−0.24	0.00	−0.23	0.00
*Saitozyma podzolica*	0.27	0.00	−0.20	0.01	−0.43	0.00	−0.43	0.00
*Microdochium* sp.	0.14	0.05	0.04	0.61	0.16	0.03	0.17	0.02
Unidentified Hypocreales	−0.26	0.00	−0.19	0.01	−0.21	0.00	−0.20	0.01
*Fusarium* sp.	−0.25	0.00	−0.08	0.31	−0.16	0.03	−0.17	0.02
*Penicillium brasilianum*	−0.11	0.14	−0.31	0.00	−0.25	0.00	−0.26	0.00
*Sarocladium kiliense*	−0.33	0.00	−0.19	0.01	−0.15	0.04	−0.16	0.03
*Penicillium simplicissimum*	0.20	0.01	0.09	0.30	0.08	0.21	0.07	0.31
*Humicola homopilata*	0.44	0.00	0.03	0.67	−0.33	0.00	−0.32	0.00
*Penicillium simplicissimum*	−0.02	0.77	−0.14	0.07	−0.13	0.07	−0.13	0.07
*Staphylotrichum* sp.	−0.04	0.64	0.01	0.83	0.21	0.00	0.24	0.00
*Apiospora malaysiana*	−0.14	0.05	−0.25	0.00	−0.13	0.07	−0.13	0.07
*Staphylotrichum* sp.	−0.05	0.50	0.06	0.46	0.24	0.00	0.27	0.00
*Solicoccozyma terricola*	0.28	0.00	−0.24	0.00	−0.46	0.00	−0.46	0.00
*Stachybotrys chartarum*	−0.18	0.01	−0.14	0.06	−0.11	0.11	−0.11	0.10

**TABLE 4:** Correlation test of relative abundances of random forest selected important bacterial taxa from the turf rhizosphere soil prior to *Clarireedia* inoculation with turfgrass greenness after disease development, and field nitrogen and fungicide applications

	Greenness (20 DAI)		N quantity		Fungicide quantity		Fungicide frequency	
Bacterial taxa	R	*P*-value	R	*P*-value	R	*P*-value	R	*P*-value
*Paenibacillus* sp.	0.00	0.98	0.11	0.17	−0.02	0.84	0.00	0.95
*Novosphingobium resinovorum*	−0.35	0.00	−0.18	0.01	−0.15	0.07	−0.13	0.10
*Massilia* sp.	−0.25	0.00	−0.09	0.24	0.16	0.06	0.15	0.07
*Arthrobacter alpinus*	−0.18	0.02	0.03	0.64	0.25	0.00	0.23	0.00
*Mesorhizobium ciceri*	0.15	0.04	0.11	0.17	0.14	0.08	0.12	0.12
*Sphingobium* sp.	0.10	0.15	0.05	0.57	0.14	0.08	0.14	0.10
*Paenibacillus agarexedens*	−0.26	0.00	−0.34	0.00	−0.16	0.06	−0.16	0.07
*Methylocella* sp.	−0.23	0.00	−0.43	0.00	−0.24	0.00	−0.26	0.00
Unidentified Fibrobacteraceae	−0.16	0.03	−0.11	0.17	0.03	0.68	0.05	0.50
*Bradyrhizobium elkanii*	0.29	0.00	0.23	0.00	0.21	0.01	0.19	0.02
*Ancylobacter* sp.	−0.34	0.00	−0.40	0.00	−0.36	0.00	−0.36	0.00
*Novosphingobium resinovorum*	−0.06	0.41	0.09	0.23	0.08	0.31	0.09	0.29
Unidentified Xanthobacteraceae	0.24	0.00	0.24	0.00	−0.01	0.84	−0.01	0.94
*Paenibacillus alginolyticus*	−0.01	0.91	−0.02	0.82	0.08	0.34	0.06	0.44
*Fontimonas* sp.	−0.16	0.03	−0.21	0.01	−0.07	0.34	−0.08	0.34
*Bdellovibrio* sp.	−0.29	0.00	−0.17	0.03	−0.07	0.37	−0.07	0.40
*Georgfuchsia* sp.	−0.22	0.00	−0.15	0.05	−0.12	0.11	−0.12	0.14
*Sphingopyxis macrogoltabida*	−0.14	0.05	0.05	0.54	0.13	0.09	0.13	0.10
*Phenylobacterium* sp.	0.14	0.05	0.24	0.00	0.14	0.08	0.15	0.07

### Linking disease suppressiveness to management practices

**Fig 5:**
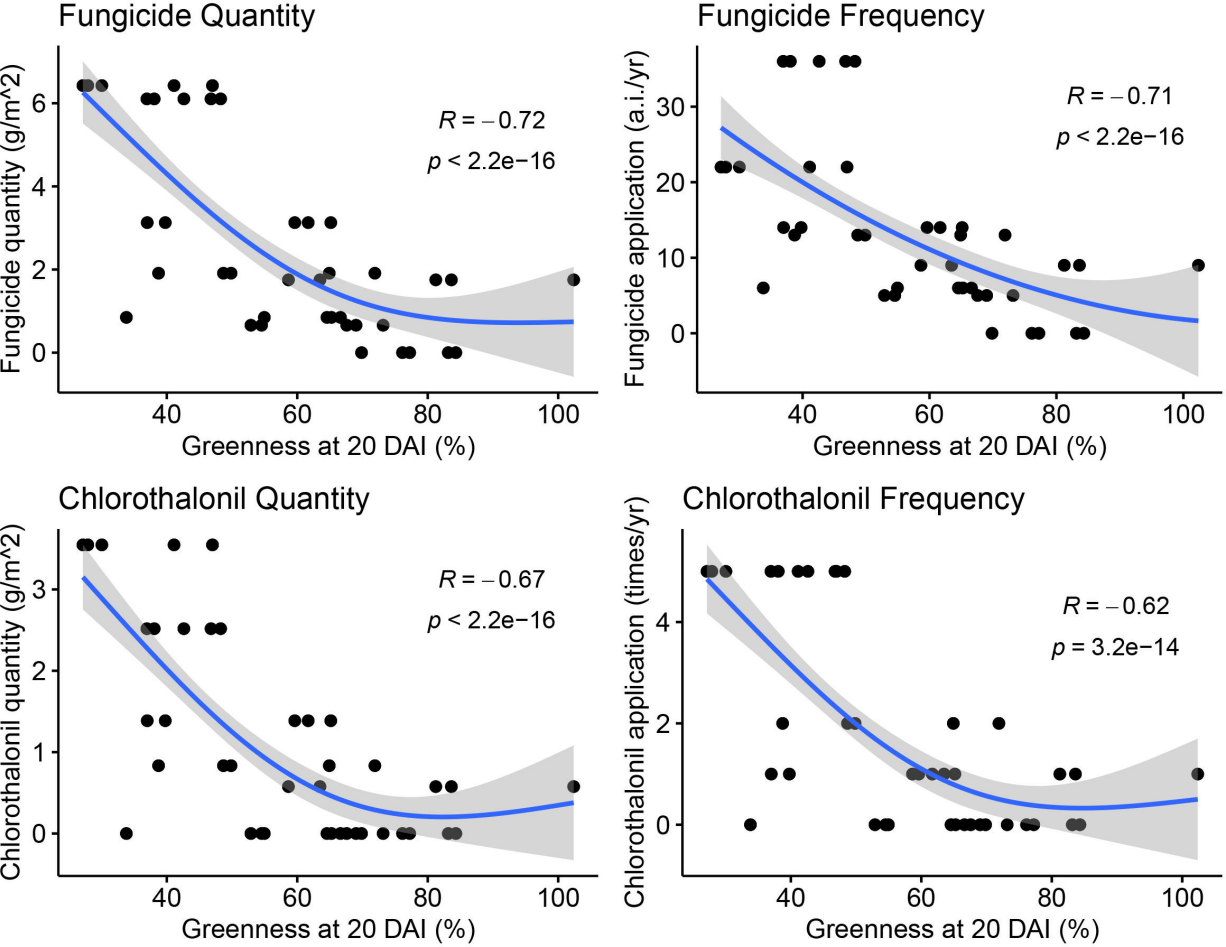
Correlation analyses of total fungicide and chlorothalonil application quantity and frequency in the field with the field microbiome transplanted turfgrass greenness, the indicator for dollar spot suppressiveness, after incubation with *Clarirdeeia jacksonii* under disease favorable condition for 20 days.

Correlation analysis showed a significant negative correlation between fungicide application intensity and dollar spot suppressiveness where both fungicide application quantity (g/m^2^) (R = −0.72, *P* < 2.2e−16) and frequency (sum of a.i. × times) (R = −0.71, *P* < 2.2e−16) were significant (Fig. 5). Total N application did not significantly correlate with dollar spot suppressiveness (R = −0.011, *P* = 0.9) (Fig. S8). To better establish the relationship between fungicide use intensity and microbiome disease suppression, we conducted a counterfactual analysis to simulate the disease outcome probability under the lowered fungicide application conditions. The statistical results suggest that we reject the hypotheses of no effect (KS-statistic *P*-value = 0.02 and CMS-statistic *P*-value = 0.01) and negative effect (KS-statistic *P*-value = 0.02 and CMS-statistic *P*-value = 0.01) but cannot reject hypotheses of constant effect (KS-statistic *P*-value = 0.66 and CMS-statistic *P*-value = 0.27) and positive effect (KS-statistic *P*-value = 0.97 and CMS-statistic *P*-value = 0.97) on the microbial disease suppression of the reduced fungicide use (Fig. S9). In addition, specific fungicides and fungicide classes were examined for their relationship with dollar spot microbial suppressiveness using partial least squares regression (PLSR). The PLSR model suggested effective prediction of turfgrass greenness at 20 DAI using all five fungicides or fungicide classes commonly used at the sampling sites (*P*-value = 8e-4). Fluazinam, among all fungicides, had the most predictive power followed by chlorothalonil, demethylation inhibitor (DMI) fungicides, dicarboximide fungicide, and succinate dehydrogenase inhibitor (SDHI) fungicides (Fig. S10).

Many important disease-suppressive microbial predictors were significantly correlated with the fungicide and nitrogen application intensity (Tables 3 and 4). The significantly correlated microbes were generally shared between fungicide application frequency and quantity. For fungicide application intensity, the fungi *Fusarium graminearum*, *Mucor moelleri*, unidentified *Hypocreales*, *Fusarium* sp., *Penicillium brasilianum*, *Sarocladium kiliense*, *Humicola homopilata*, and *Solicoccozyma terricola* (Table 3) and the bacteria *Methylocella* sp. and *Ancylobacter* sp. were negatively correlated (Table 4). In contrast, the fungi *Microdochium* sp. and *Staphylotrichum* sp. and the bacteria *Arthrobacter alpinus* and *Bradyrhizobium elkanii* were positively correlated with fungicide application intensity (Table 3). Although nitrogen application did not correlate with disease suppressiveness, significant correlations with relative abundances of individual taxa were observed. Fungi *Microdochium neoqueenslandicum,* bacteria *Bradyrhizobium elkanii*, unidentified Xanthobacteraceae, and *Phenylobacterium* sp. positively correlated with the N application, whereas fungi *Fusarium graminearum*, *Saitozyma podzolica*, unidentified Hypocreales, *Penicillium brasilianum*, *Sarocladium kiliense*, *Apiospora malaysiana*, *Solicoccozyma terricola,* and bacteria *Novosphingobium resinovorum*, *Paenibacillus agarexedens, Methylocella* sp., *Ancylobacter* sp., *Fontimonas* sp., *Bdellovibrio* sp., and *Georgfuchsia* sp. were negatively correlated with the N application (Tables 3 and 4).

## DISCUSSION

### Presence of disease-suppressive soil in turfgrass

We observed differences in microbiome-mediated dollar spot suppressiveness among soils collected from 14 locations across the Midwest and Northeast U.S. encompassing golf courses, agricultural, and native prairie landscapes. To our knowledge, this is the first description of disease-suppressive soils in turfgrass. We also demonstrated that the suppressive ability of the soil could be transplanted to a sterile potting media, which is typically a key aspect of specific suppressive soils and may provide future directions for research in turfgrass and other agricultural and horticultural pathosystems. Conferring disease suppression by transplanting a disease-suppressive soil into a conducive soil has been observed in several plant pathosystems including *Rhizoctonia solani* in sugar beet (53), and *Ralstonia solanacearum* in eggplant and tomato (54, 55). In all these studies, the primary mechanism for disease suppression was found to be the retention and enrichment of microbes antagonistic to the pathogen. Although this is yet to be verified in our study, the observed disease suppression translatability may suggest the enrichment of plant growth-promoting microbes and microbes antagonistic to *Clarireedia*.

Another interesting finding is that turf grown with the forest soil microbiome had a lower dollar spot severity than turf grown with the prairie soil microbiome. In fact, turf transplanted with the prairie microbiome had higher dollar spot severity than all other treatments except for one (NE-High2), suggesting potential dysbiosis after transplant of prairie soil microbiome. It is also possible that the *Clarireedia* had better fitness in the prairie-associated microbial community than that of forest as sampled prairies were likely to have more colonization of Poaceae species, which is phylogenetically close to the hosts of *Clarireedia*. Additionally, forest soil may harbor microbes that suppress fungal pathogens or promote creeping bentgrass defense against dollar spot. Multiple studies have found forest soil harboring antifungal-producing microbes or those that can induce systemic disease resistance (56–58).

### Fungicide intensity impacts dollar spot suppressiveness

There was a clear inverse relationship in our study between fungicide intensity at the sampling location and dollar spot suppressiveness in the inoculated pot assay. Furthermore, the use of contact fungicides including fluazinam and chlorothalonil seemed to have more impact on the microbial *Clarireedia* suppressiveness as they were identified as top turf greenness predictors at 20 DAI. Specific and induced disease-suppressive soil formation depends largely on soil management and favors management that allows for the growth, selection, and coevolution of the plant, antagonistic microbes, and the pathogen in a monocropping system over a relatively long time scale (7). Past research has found that amendments that enriched the soil microbial diversity, such as organic soil amendments, were found to effectively confer disease suppression (59, 60). In turfgrass, compost applications were shown to suppress dollar spot in turfgrass with a postulated mechanism of reduced pathogen fitness due to microbial competition (61). However, the role of fungicide applications in suppressive soil formation has remained unclear. This present study demonstrates that reduced fungicide intensity can also induce disease-suppressive soil as it potentially allows a more diverse soil microbiome to grow and facilitate the plant-microbe and microbe-microbe coevolution between plant, antagonistic microbes, and the pathogen. More specifically, fewer chemical inputs contributing to increased dollar spot suppressiveness supports the proposed theoretical framework of induced suppressive soil formation in response to pathogen activity (8).

Golf courses with lower fungicide use intensity generally had higher levels of dollar spot suppression. However, some golf courses with lower fungicide intensity failed to sustain dollar spot suppression after microbiome transplanting throughout the entire controlled environment study. This likely is an indicator of natural, rather than induced, disease suppression, which is largely dependent on the soil’s physical and chemical properties (7). In addition, loss of microbial diversity, especially for the fungal community, due to freezing of field soil inocula in storage may have also led to failed disease suppression in some of the treatments and may explain why fungal predictors accounted for less variance in the RF-suppressive soil predictive model. Another explanation may be that the controlled environment and the newly established turf in our study provided low fitness for the key plant beneficial and pathogen-antagonistic microbes to colonize and thrive, thus failing to provide the plant beneficial functions. Nevertheless, with the observed link between field fungicide usage and induced disease suppression in a controlled environment, this study reveals the essential role of fungicide application in disease-suppressive soil formation.

### Potential dollar spot inhibitory and turf health-promoting microbes in the soil

The RF models identified several microbes that were positively correlated with dollar suppressiveness and negatively correlated with fungicide intensity at pathogen inoculation. These included fungi such as *Mucor moelleri*, *Humicola homopilata*, and *Solicoccozyma terricola. Mucor moelleri* has been shown to promote plant growth through antagonistic activity against the fungal pathogens *Athelia rolfsii* and *Colletotrichum gloeosporiodes* in both infested tomato plants and using *in vitro* assays (62). Many species in the genus *Chaetomium* were previously found to produce diverse bioactive compounds including many antibiotics (63) and have been suggested as a potential biocontrol agent against a broad spectrum of plant oomycetes and fungal pathogens such as *Phytophthora* sp. in durian, black pepper, and tangerine; *Fusarium oxysporum* in tomato; and *Sclerotium rolfsii* in corn (64). *Solicoccozyma terricola* is linked to soil biomass degradation and was previously found to be enriched in *Streptomyces lydicus* M01-treated soil for *Alternaria* leaf spot suppression in cucumber (65).

Bacteria identified by the RF models that were positively correlated with dollar spot suppression were *Mesorhizobium ciceri*, *Bradyrhizobium elkanii*, unidentified *Xanthobacteraceae*, and *Phenylobacterium* sp. Among the four bacteria that were positively correlated with higher dollar spot suppressiveness, two of them were root-nodulating bacteria that have proven plant growth promotional effects. *Mesorhizobium ciceri* was found to facilitate nutrient acquisition and also alleviate the negative effect of fungicide kitazin on Chickpea (*Cicer aritienum* L.) with reduced oxidative damage and cell death (66). *Bradyrhizobium elkanii* is a well-studied symbiont with many legume species that fix atmospheric N into plant available N and facilitates increased plant growth (67, 68) and was found to increase cowpea growth under water deficit scenarios (69). The higher relative abundance of *Phenylobacterium* sp. was previously associated with improved barley growth, but the actual plant growth-promoting mechanism remains unclear (70). Future research on dollar spot suppressive soils and plant health-promoting microbes should focus on these organisms because of their strong correlation with reduced dollar spot in this study.

### Potential dollar spot inhibitory and turf health-promoting microbes in the phyllosphere

The field soil transplant also contributed to the phyllosphere microbiome assembly at pathogen inoculation. Important fungal predictors of disease suppression selected by the RF model included many known antifungal compound-producing fungi such as *Cladosporium sphaerospermum* ASVs, *Sarocladium kiliense*, *Microdochium* sp., *Penicillium simplicissimum*, *Staphylotrichum* sp., and *Alternaria* sp. Almost all RF-selected bacteria in the phyllosphere were previously shown to have antifungal properties, including notable ones such as *Stenotrophomonas* sp. and *Paenarthrobacter* sp.

The bacterium *Stenotropphomonas rhizophila* was identified as the most important phyllosphere bacterial predictor by the RF model. This bacterium was previously found to have antifungal properties, inhibited the growth of plant pathogens *Alternaria alternata* and *Botrytis cinerea in vitro*, and suppressed *B. cinerea* infection when sprayed on tomato leaves (71, 72). *Paenarthrobacter* sp. was found to be the plausible key taxa in a *Rhizoctonia solani*-suppressive soil in rice where *in vitro* experiments using the bacterial isolate from the suppressive soil confirmed the suppression activity of *P. ureafaciens* against fungal phytopathogens such as *R. solani* and *Colletotrichum* sp (73). Although there are known species of *Microdochium* that can cause disease in turfgrass, namely *Microdochium nivale*, many other *Microdochium* species can produce antifungal compounds such as isocoumarin derivatives and monocerin (74) and have shown biocontrol activity in barley against *Gaeumannomyces graminis* var. tritici (75). Species in *Staphylotrichum* were found to mildly inhibit the growth of the plant pathogen *Corynespora cassiicola* on PDA *in vitro* (76) and was associated with healthy Ginseng (*Panax notoginseng*) in soil conducive to replant root rot (77). *Penicillium simplicissimum* can produce diverse antifungal compounds (78, 79) and induce plant systemic defense response in cucumber (*Cucumis sativus*) against *Colletotrichum orbiculare* (80). It has also demonstrated efficacy as a biological control agent against *Pythium* damping-off in beetroot (*Beta vulgaris*) (81), *Verticillium* wilt in cotton (*Gossypium hirsutum*) (82), and *Puccinia striiformis* f. sp. *tritici* in wheat (83). *Cladosporium sphaerospermum* produces diverse polyketides that have shown biological control effects against Botrytis *in vitro* and on strawberry and tomato fruits *in vivo* (84, 85). The red sage endophytic fungus *Sarocladium kiliense* has been shown to produce antifungal compounds that were confirmed with an *in vitro* plate assay (86).

All of these organisms were found either in higher abundances or as effective dollar spot-suppression predictors in the phyllosphere of plants grown in soils from sites with reduced fungicide usage, suggesting a connection between the soil microbiome and phyllosphere that can mediate disease development. In addition, our findings also support the previous studies on soil as a source for phyllosphere microbiome assembly, which provides essential functions such as pathogen suppression (87–89).

### Untargeted effect of fungicide may modulate plant-beneficial microbes

The counterfactual and correlation analyses indicated a link between reduced fungicide use intensity and improved microbial disease suppression. The PLSR model further suggested that contact fungicides such as fluazinam and chlorothalonil may have more influence on the microbiome disease suppressiveness than penetrant fungicides. Fluazinam (3-chloro-N-(3-chloro-2,6-dinitro-4-(trifluoromethyl)phenyl)−5-(trifluoromethyl)−2-pyridinamine) is a pyridinamine fungicide with a suggested mode of action being inhibition of ATP synthetase in fungal cells (90). Chlorothalonil (2,4,5,6-tetrachloroisophthalonitrile) is a chloronitrile fungicide that reacts with fungal thio-dependent enzymes and leads to cell death (91). Both chlorothalonil and fluazinam are commonly used to control a broad array of turfgrass foliar diseases. Their impact on the turfgrass microbiome has not been investigated but was previously found to impact the microbiome of other cropping systems (18, 92–94). In addition to the fungicidal effect of fluazinam, it was found to be highly toxic to bacteria in the luminescent bacteria toxicity test in a controlled environment and a potato field soil that received 205 g/ha (92). In the same study, the authors also found that the residue of fluazinam is long lasting, as the transformation products can be found throughout the season and even over-winter in the soil (92). In one study, the application of fluazinam in Chinese cabbage (*Brassica rapa*) led to reduced fungal abundance and short-term elevated bacterial diversity, and the associated catabolism functional diversity likely due to increased substrate availability from fungal death (95). When compared with the contact fungicide mancozeb, fluazinam impacted the soil bacteria to a lesser extent but still eliminated 25 bacterial species from 0.95 L of soil received 0.054 g of Fluazinam after 6 weeks (94).

Chlorothalonil is known to have bactericidal effects and is used to control several bacterial diseases in plants suggesting its cross-kingdom broad-spectrum activity (96). Application of chlorothalonil was previously found to significantly affect soil bacterial and fungal community structure and inhibit soil dehydrogenase, catalase, and acid phosphatase activities (18, 93). Interestingly, a recent study found that chlorothalonil suppressed the pathogen-antagonistic bacteria on amphibian (*Lithobates vibicarius*) skin, which could potentially lead to the loss of host immunity (97). Although the results from our study suggest that fluazinam and chlorothalonil can reduce the activity of microbes antagonistic to *Clarireedia* spp., direct research exploring these potential effects in greater detail is warranted to understand the mechanisms behind the effects.

Other indirect effects of fungicides on soil ecosystem functions, microbial diversity nutrient cycling, and disease susceptibility have been extensively studied and reviewed in the past (70, 98). For example, X. Wang et al. (17) showed that application of the quinone outside inhibitor (QoI) fungicide azoxystrobin inhibited the activities of urease, invertase, and phosphatase while promoting the activity of catalase in the soil. In this same study, bacterial α-diversity was reduced, and the community was restructured by azoxystrobin. Repeated fungicide applications can also reduce the colonization of arbuscular mycorrhizal fungi (AMF) (15) and decrease the activity of root nodule-forming bacteria (99). In contrast, one study found that foliar application of the QoI fungicide pyraclostrobin enhanced root nodulation and nitrogen fixation in soybeans. Bacteria-formed root nodules host a variety of plant-beneficial microbes that release plant hormones, facilitating nutrient uptake, and many of them can also produce antifungal compounds (100), which potentially protect plants from fungal pathogens.

For the untargeted effect of fungicides specific to our study, among the bacteria indicated as important by the RF model, *Ancylobacter* sp. and *Methylocella* sp. negatively correlated with fungicide intensity. Interestingly, both bacteria negatively correlated with the fungicide intensity were methylotrophic (101, 102). Species in the genus *Ancylobacter* were found to fix N and promote the growth of rice (103) and can potentially regulate the ethylene signaling, providing N and phosphate to plants according to *in vitro* substrate utilization assays (104). Also, many species in *Ancylobacter* are capable of utilizing oxalate as a carbon source (105, 106) , which has been identified as one of the major virulence factors in *Clarireedia* (107, 108). Therefore, *Ancylobacter* might be another crucial organism that played a role in suppressing dollar spot severity in this study. Collectively, these results suggest that higher fungicide application intensity may be negatively impacting plant-beneficial bacteria and fungi in the soil, leaving the turfgrass plant more susceptible to disease development.

It is worth mentioning that the analyses in this study do not suggest a causative link between fungicides and microbiome alterations but rather statistically supported inferences on their association. Thus, no fungicide recommendation or environmental concerns should be raised until further research can be conducted. Also, to reiterate many previous studies on the importance of long-term study on the effects of microbiome manipulation (7, 8, 109, 110), long-term studies on fungicide effects in comparison of multiple sites with control plots are warranted.

## Data Availability

Amplicon sequence data and the metadata collected are available in the Sequence Read Archive under BioProject accession numbers PRJNA1002783 and PRJNA1002784.

## SUPPLEMENTAL MATERIAL

Supplemental figures aem.01771-24-s0001.docx Figures S1 to S10.

Supplemental tables aem.01771-24-s0002.docx Tables S1 and S2.

ASM does not own the copyrights to Supplemental Material that may be linked to, or accessed through, an article. The authors have granted ASM a non-exclusive, world-wide license to publish the Supplemental Material files. Please contact the corresponding author directly for reuse.
