# Supplementary material for: Fungicide use intensity influences the soil microbiome and links to fungal disease suppressiveness in amenity turfgrass
Source: Appl Environ Microbiol. 2025 Feb 21;91(3):e01771-24. doi: 10.1128/aem.01771-24 (PMC11921360; doi:10.1128/aem.01771-24)
Supplement: Supplemental figures — Figures S1 to S10. [file aem.01771-24-s0001.docx]

Supplementary tables for manuscript titled:

Fungicide use intensity influences the soil microbiome and links to fungal disease suppressiveness in amenity turfgrass

Ming-Yi Chou^a,b^, Apoorva Tarihalkar Patil^a^, Daowen Huo^a^, Qiwei Lei^a^, Jenny Kao-Kniffin^c^ and Paul Koch^a^

^a^ Department of Plant Pathology, University of Wisconsin-Madison, Madison, WI 53706, USA

^b^ Department of Plant Biology, Rutgers University, New Brunswick, NJ 08901, USA

^c^ Horticulture Section, School of Integrative Plant Science, Cornell University, Ithaca, NY 14853, USA


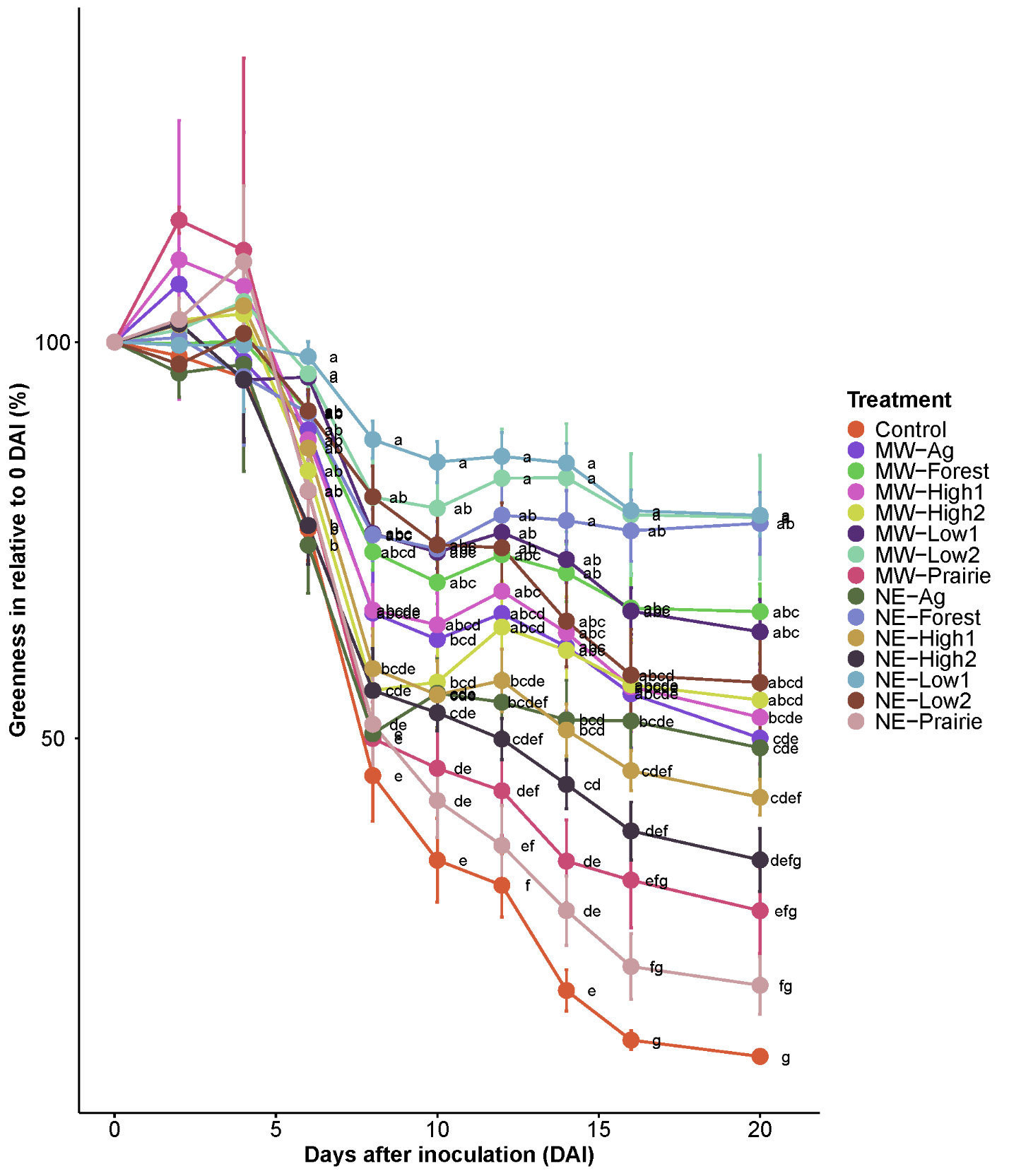


Figure S1. Decrease of turfgrass greenness as the indictor of dollar spot development after *Clarireedia* inoculation. Letters indicates the statistical difference yield from Tukey’s HSD for each day at α equals to 0.05 where no sharing letters means significantly different.


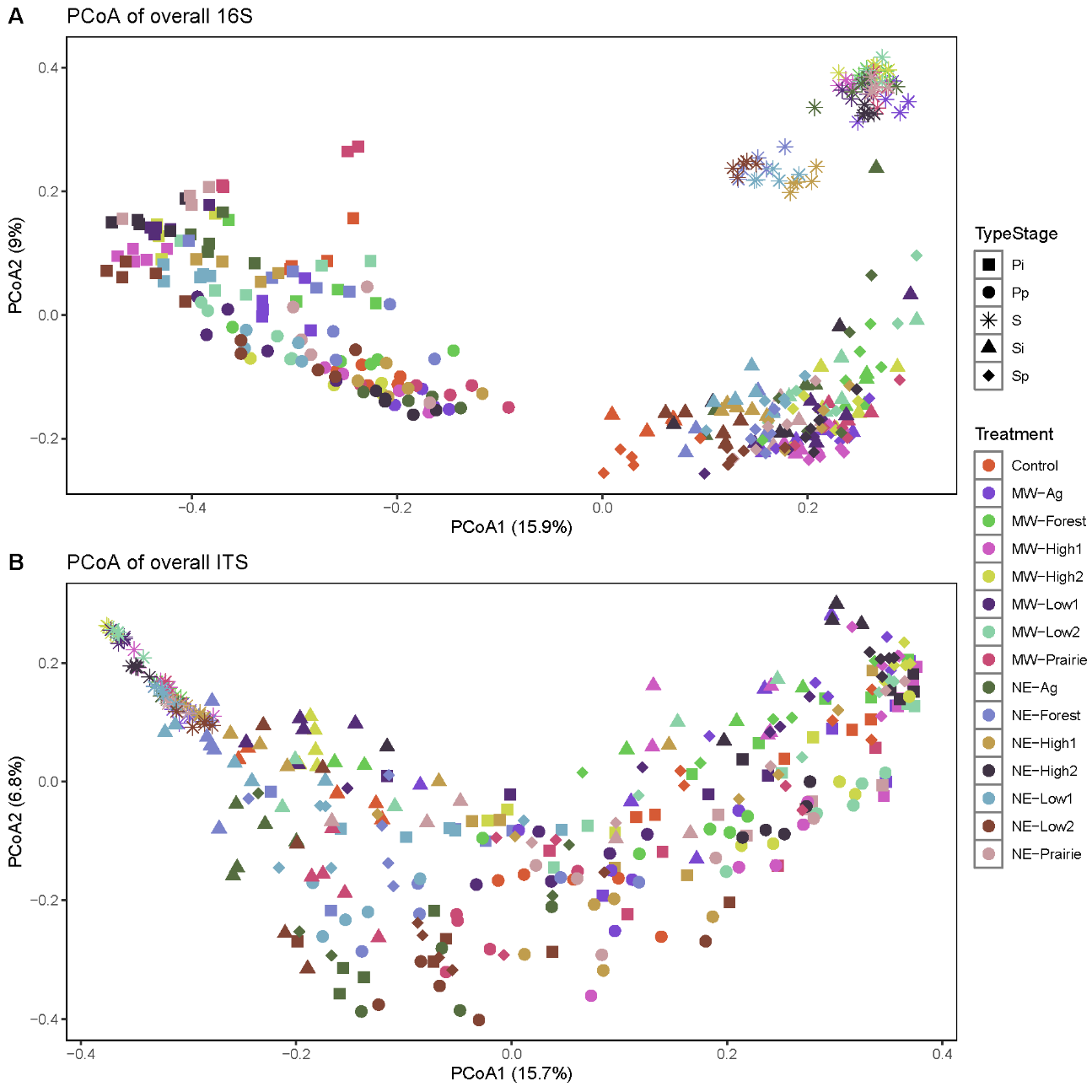


Figure S2. Principal coordinates analyses (PCoA) of microbiome associated with turfgrass grown with field microbiome transplantation presented with top two dimensions that explained most variances for all samples. The shapes indicate the sample types and sampling stages, and the colors indicate the treatments (field microbiome sources). TypeStage indicates the sample types including phyllosphere (capital P) and root-associated soil (capital S) as well as sampling stages including field inocula (no lowercase designation), pre-inoculation of pathogen (lowercase i), and peak of disease (lowercase p).


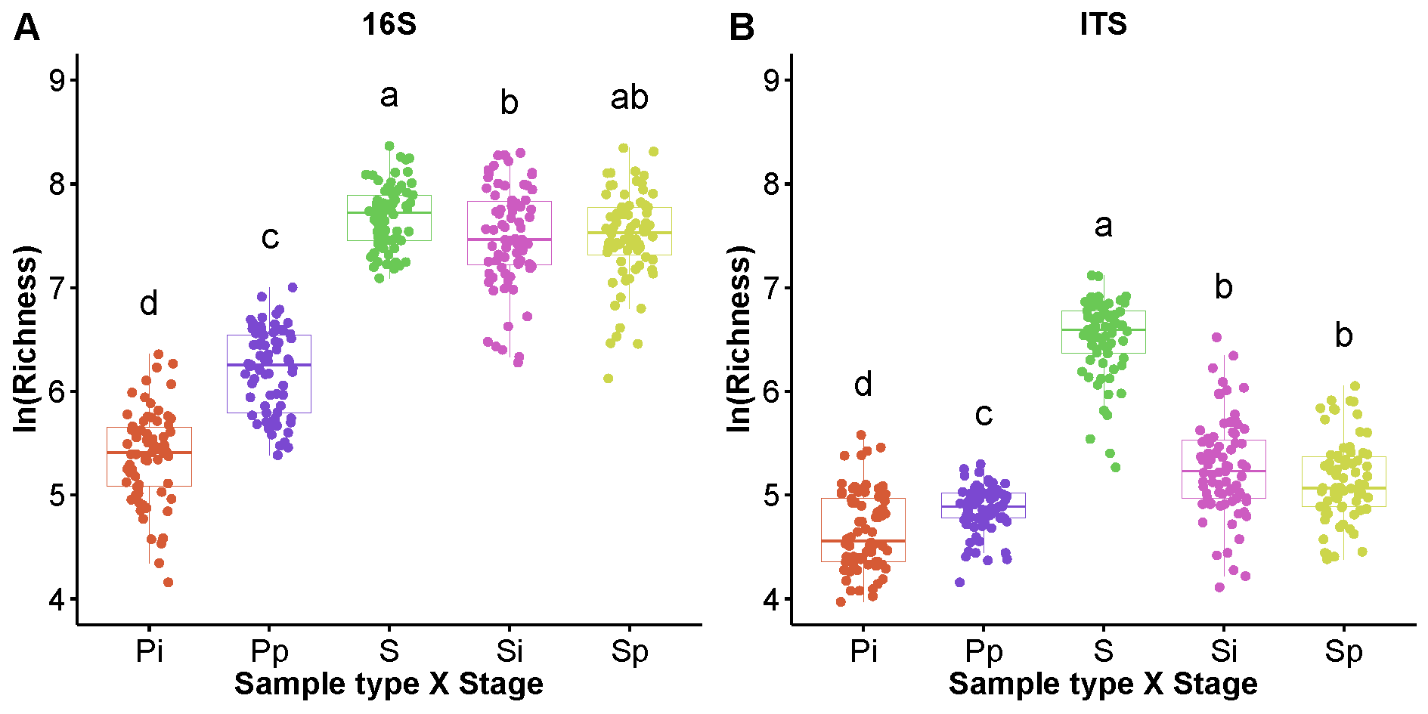


Figure S3. Boxplot showing bacterial (a) and fungal (b) ASV natural log richness. The horizonal line in each box represents median, and the upper and lower boundary of the box represents first and third quartile, respectively. Letters indicates the statistical difference yield from Tukey’s HSD for each day at α equals to 0.05 where no sharing letters means significantly different.


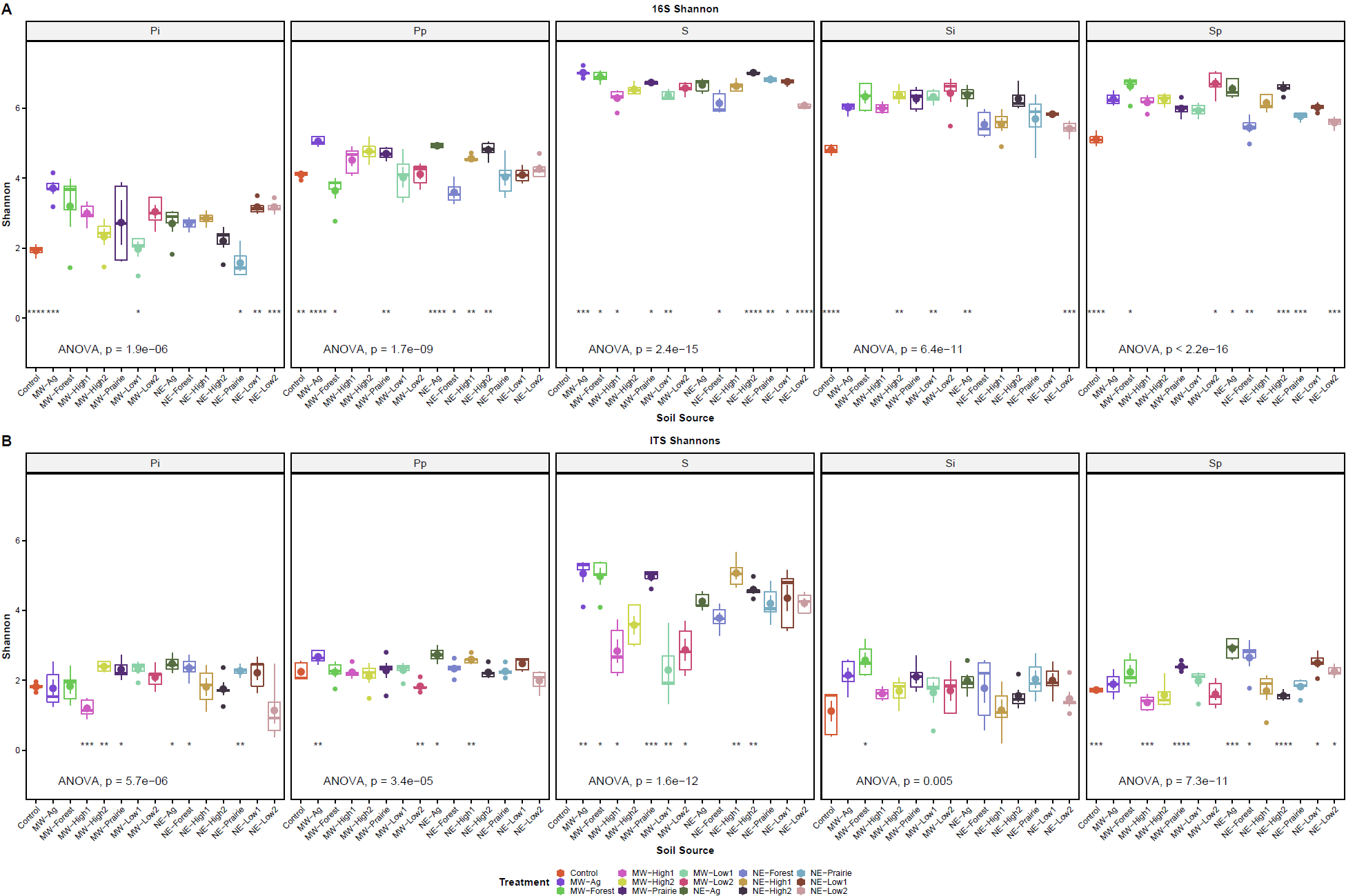


Figure S4. ASV Shannon diversity for bacterial (a) and fungal (b) communities in turfgrass grown with different sources of transplanted field microbiomes. The asterisks indicate significant mean separation derived from T-test: *, P < 0.05; **, P < 0.01; ***, P < 0.001; ****, P < 0.0001.


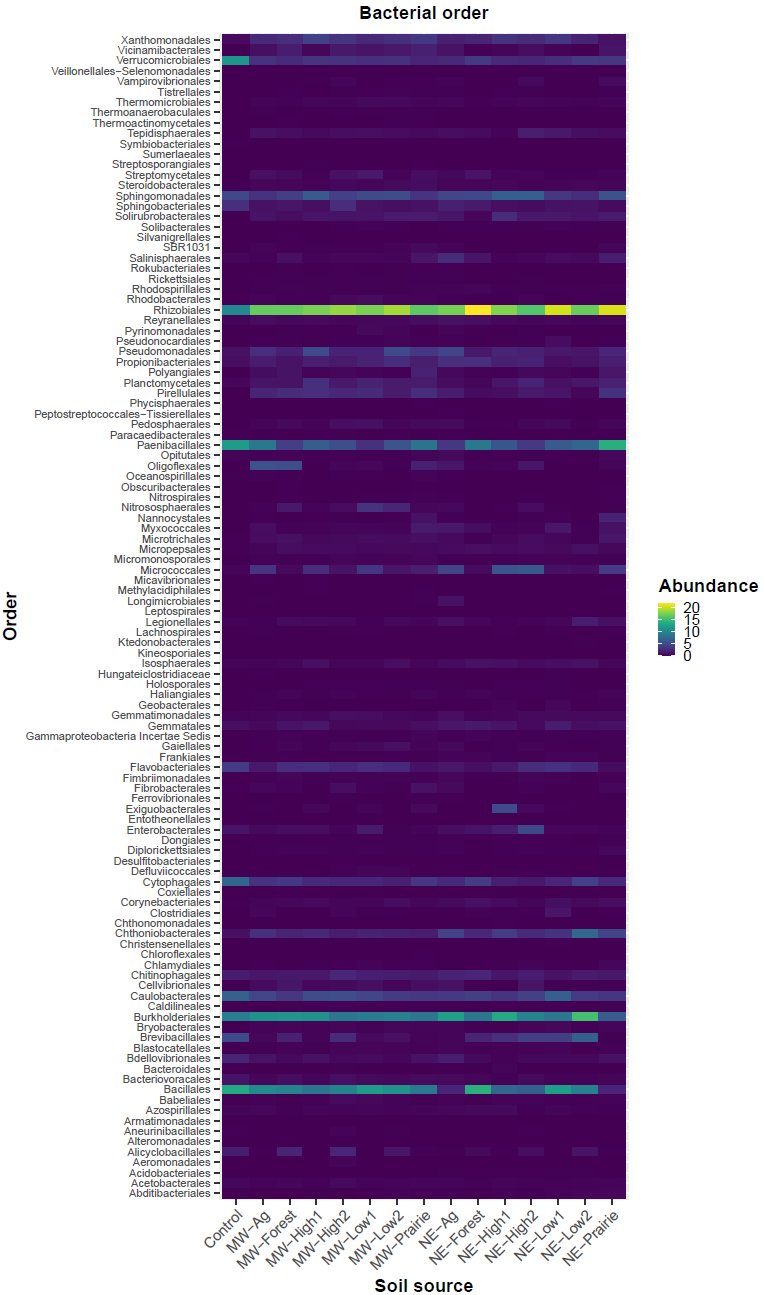


Figure S5. Heat maps showing the relative abundances of the bacterial orders of the microbiome transplanted turfgrass rhizosphere soil at *Clarireedia* inoculation.


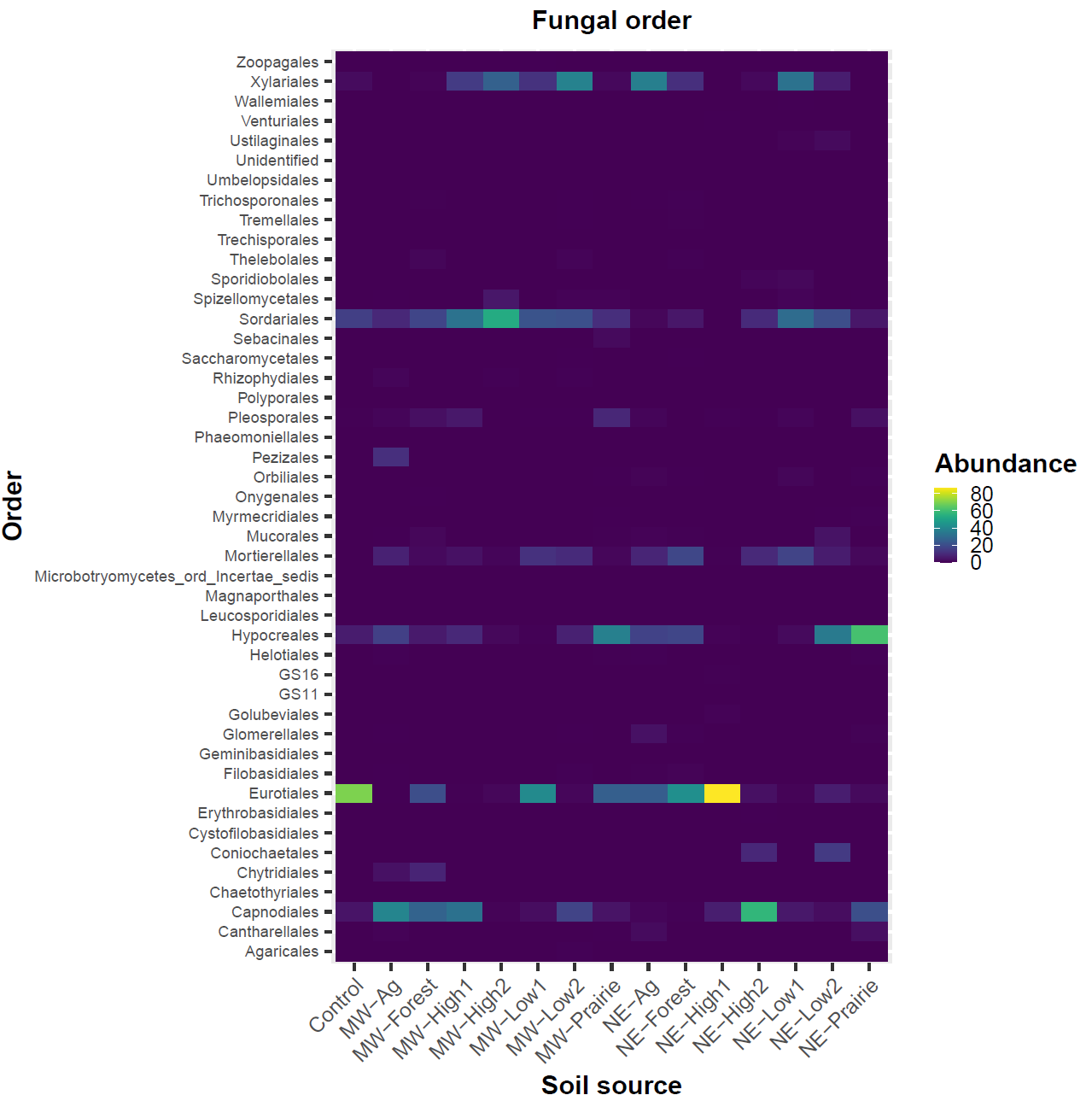


Figure S6. Heat maps showing the relative abundances of the fungal orders of the microbiome transplanted turfgrass rhizosphere soil at *Clarireedia* inoculation.


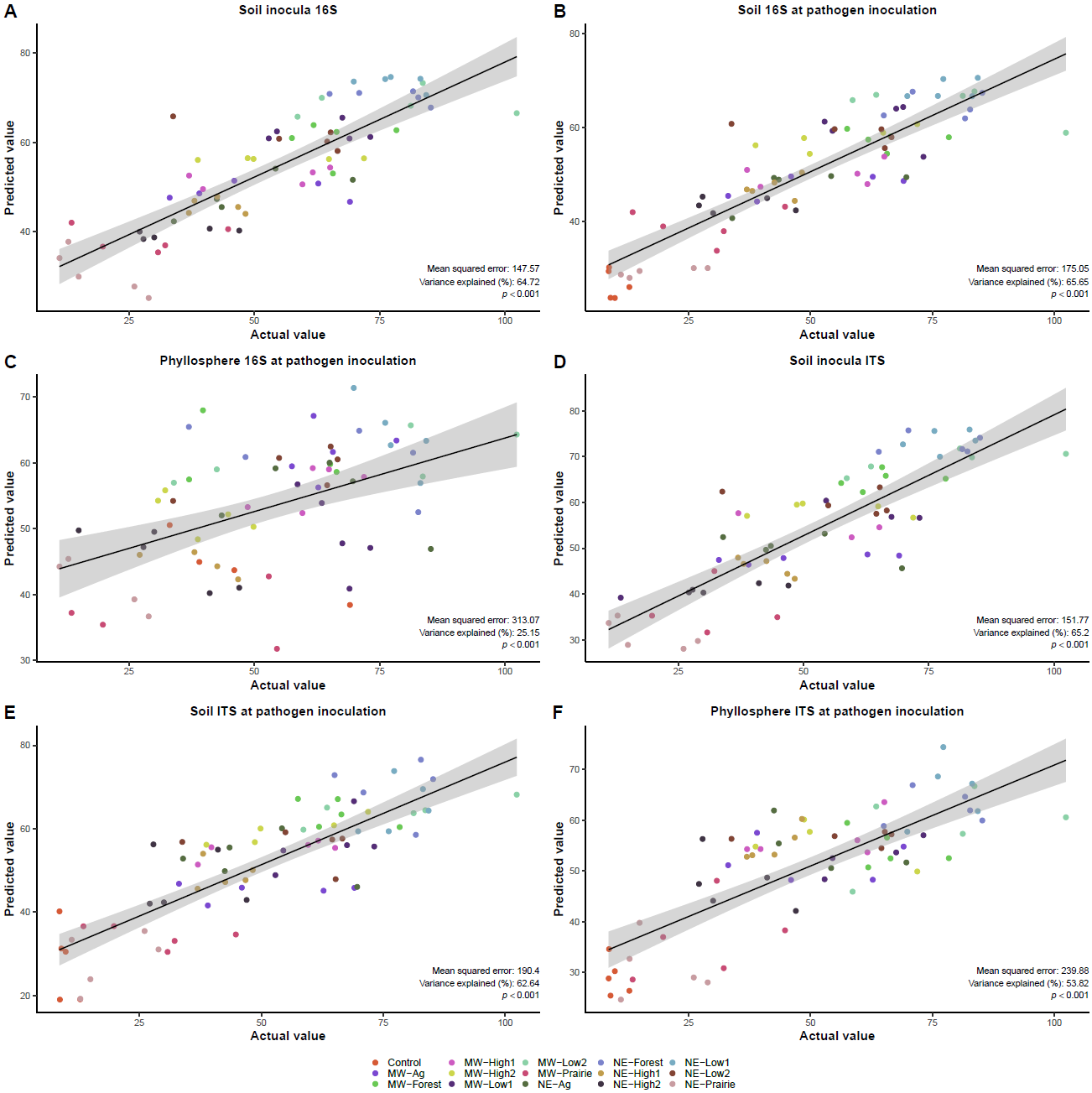


Figure S7. Random forest prediction lines by using the selected bacterial (a, b and c) and fungal (d, e and f) predictors to predict the turfgrass greenness (disease suppressiveness).


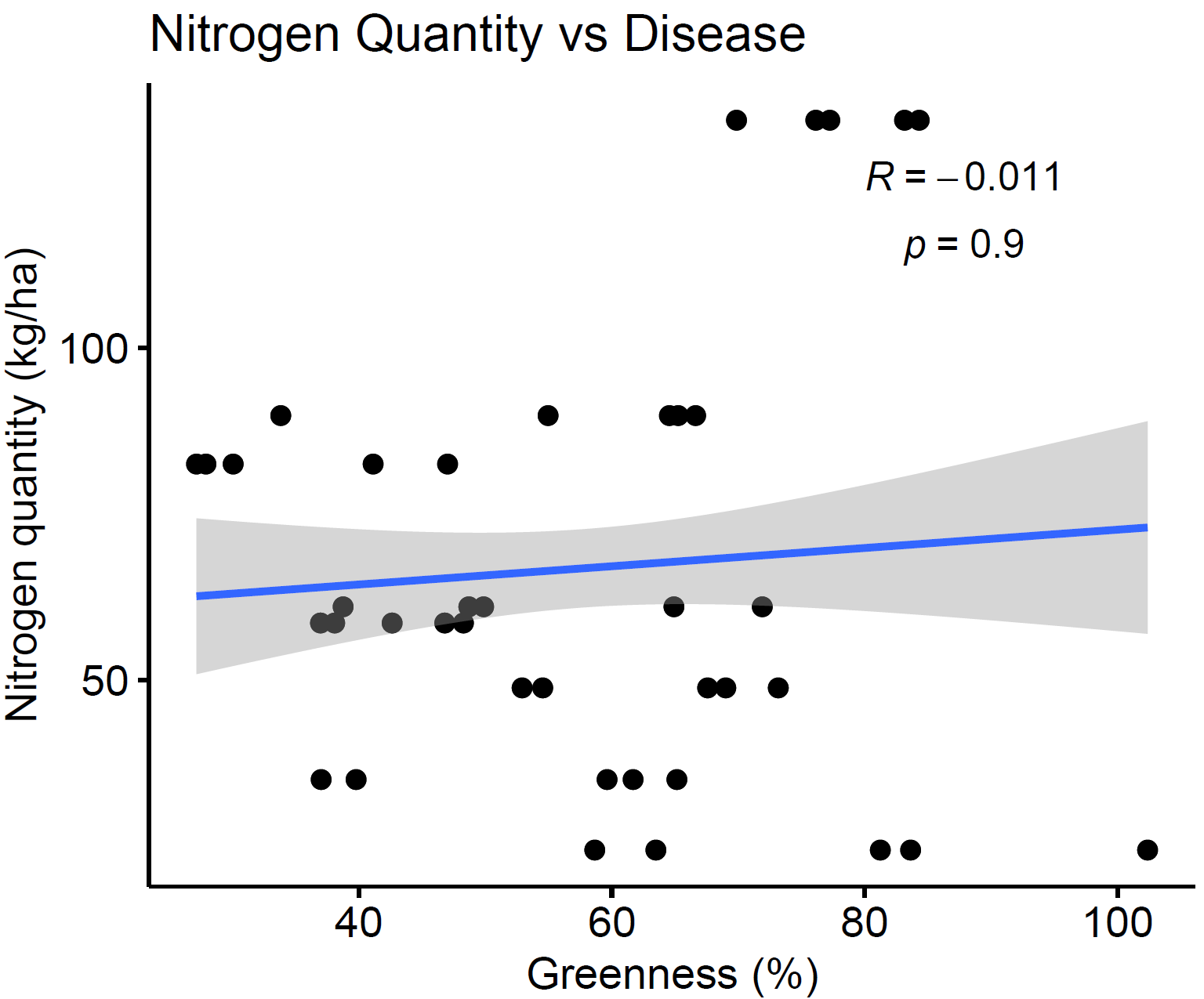


Figure S8. Correlation analysis of N application quantity in the field with the field microbiome transplanted turfgrass greenness, the indicator for dollar spot suppressiveness, after incubation with *Clarirdeeia* under disease favorable condition for 20 days.


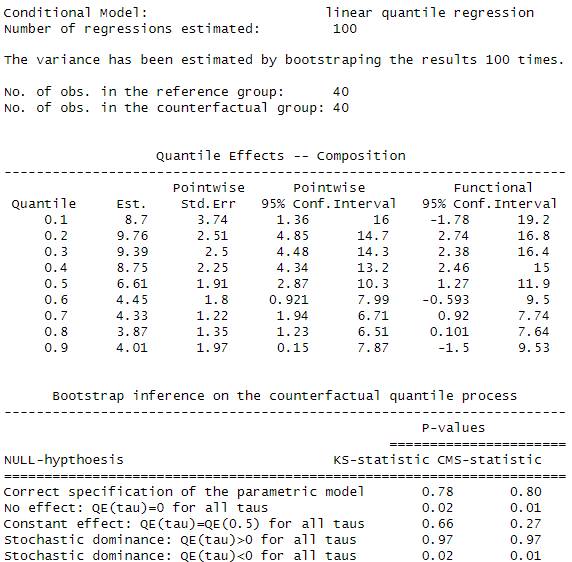


Figure S9. Counterfactual analysis of fungicide use intensity effect on the microbiome disease suppressiveness using a reduced fungicide intensity distribution as the counterfactual condition.


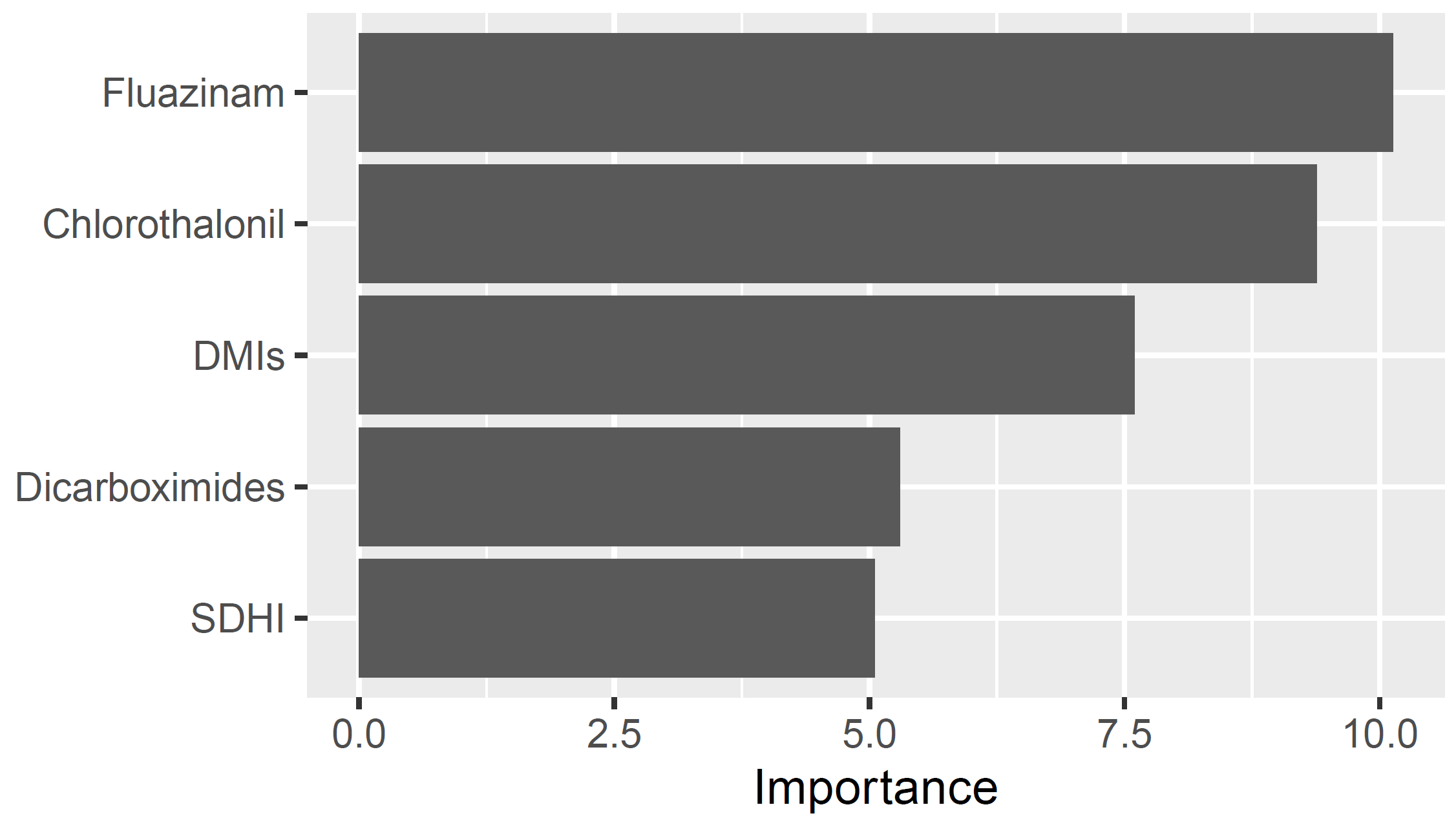


Figure S10. Importance rank of major fungicide used in predicting dollar spot suppressiveness in the Partial Least Squares regression model.
