# Supplementary material for: Fungicide use intensity influences the soil microbiome and links to fungal disease suppressiveness in amenity turfgrass
Source: Appl Environ Microbiol. 2025 Feb 21;91(3):e01771-24. doi: 10.1128/aem.01771-24 (PMC11921360; doi:10.1128/aem.01771-24)
Supplement: Supplemental tables — Tables S1 and S2. [file aem.01771-24-s0002.docx]

Supplementary tables for manuscript titled:

Fungicide use intensity influences the soil microbiome and fungal disease suppressiveness in amenity turfgrass

Ming-Yi Chou^a,b^, Apoorva Tarihalkar Patil^a^, Daowen Huo^a^, Qiwei Lei^a^, Jenny Kao-Kniffin^c^ and Paul Koch^a^

^a^ Department of Plant Pathology, University of Wisconsin-Madison, Madison, WI 53706, USA

^b^ Department of Plant Biology, Rutgers University, New Brunswick, NJ 08901, USA

^c^ Horticulture Section, School of Integrative Plant Science, Cornell University, Ithaca, NY 14853, USA

| Sample | F value | Pr(>F) |
| --- | --- | --- |
| 16S SS | 1.8587 | 0.05596 |
| ITS SS | 4.352 | 5.45E-05 |
| 16S Si | 0.5432 | 0.8968 |
| ITS Si | 1.7506 | 0.06882 |
| 16S Pi | 1.0178 | 0.45 |
| ITS Pi | 1.721 | 0.07651 |

Table S1. Bacterial and fungal ASV dispersion test of turfgrass phyllosphere and rhizosphere soil samples.

Table S2. Non-significant pairs of paired-PERMANOVA analyses for bacterial and fungal communities of field microbiome transplanted turfgrass. The pairs not shown in this table all have adjusted p-value less than the significant threshold 0.05. P-values were adjusted for multiple comparison with Benjamini-Hochberg correction.

| **Phyllosphere 16S at pathogen inoculation** | | |
| --- | --- | --- |
| Pairs | R^2^ | Adjusted p-value |
| Control vs NE-High1 | 0.35 | 0.09 |
| MW-High1 vs NE-Ag | 0.20 | 0.09 |
| MW-High2 vs MW-Low1 | 0.27 | 0.06 |
| MW-Prairie vs NE-Ag | 0.24 | 0.08 |
| MW-Prairie vs NE-Prairie | 0.30 | 0.09 |
| MW-Low1 vs NE-Prairie | 0.20 | 0.17 |
| NE-Ag vs NE-Prairie | 0.25 | 0.08 |
|  |  |  |
| **Rhizosphere soil ITS at pathogen inoculation** | | |
| Pairs | R^2^ | Adjusted p-value |
| MW-Ag vs NE-High2 | 0.16 | 0.11 |
| MW-High1 vs MW-Low2 | 0.19 | 0.09 |
| MW-High2 vs MW-Low2 | 0.15 | 0.20 |
| MW-Low1 vs MW-Low2 | 0.15 | 0.13 |
|  |  |  |
| **Phyllosphere ITS at pathogen inoculation** | | |
| Pairs | R^2^ | Adjusted p-value |
| MW-High2 vs MW-Low1 | 0.22 | 0.06 |
| MW-High2 vs MW-Low2 | 0.17 | 0.20 |
| MW-Prairie vs MW-Low2 | 0.22 | 0.05 |
| MW-Prairie vs NE-Prairie | 0.19 | 0.11 |
| MW-Low1 vs MW-Low2 | 0.18 | 0.18 |
|  |  |  |
| **Phyllosphere ITS at peak disease** | | |
| Pairs | R^2^ | Adjusted p-value |
| MW-High2 vs MW-Low2 | 0.24 | 0.05 |
| MW-High2 vs NE-High2 | 0.18 | 0.07 |
| NE-Ag vs NE-Forest | 0.21 | 0.05 |
